# Apolipoprotein A-V is a potential target for treating coronary artery disease: evidence from genetic and metabolomic analyses

**DOI:** 10.1016/j.jlr.2022.100193

**Published:** 2022-03-10

**Authors:** Dorina Ibi, Manon Boot, Martijn E.T. Dollé, J. Wouter Jukema, Frits R. Rosendaal, Constantinos Christodoulides, Matt J. Neville, Robert Koivula, Patrick C.N. Rensen, Fredrik Karpe, Raymond Noordam, Ko Willems van Dijk

**Affiliations:** 1 Department of Human Genetics, Leiden University Medical Center, Leiden, The Netherlands; 2 Department of Public Health and Primary Care, National Institute for Public Health and the Environment (RIVM), Bilthoven, The Netherlands; 3 Department of Cardiology, Leiden University Medical Center, Leiden, The Netherlands; 4 Netherlands Heart Institute, Utrecht, The Netherlands; 5 Department of Clinical Epidemiology, Leiden University Medical Center, Leiden, The Netherlands; 6 Oxford Centre for Diabetes, Endocrinology and Metabolism, Radcliffe Department of Medicine, University of Oxford, Oxford, United Kingdom; 7 NIHR Oxford Biomedical Research Centre, Oxford University Hospitals Foundation Trust, Oxford, United Kingdom; 8 Division of Endocrinology, Department of Internal Medicine, Leiden University Medical Center, Leiden, The Netherlands; 9 Einthoven Laboratory for Experimental Vascular Medicine, Leiden University Medical Center, Leiden, The Netherlands; 10 Division of Gerontology and Geriatrics, Department of Internal Medicine, Leiden University Medical Center, Leiden, The Netherlands

**Keywords:** cardiovascular disease, lipoproteins, triglycerides, lipid-lowering therapy, hyperlipidemia, LDL, genetic variation, lipoprotein lipase, factorial analysis, clinical data, apo A-V, apolipoprotein A-V, apo B, apolipoprotein B, GRS, genetic risk scores, NEO, Netherlands Epidemiology of Obesity, OBB, Oxford Biobank

## Abstract

Triglyceride (TG)-lowering *LPL* variants in combination with genetic LDL-C-lowering variants are associated with reduced risk of coronary artery disease (CAD). Genetic variation in the *APOA5* gene encoding apolipoprotein A-V also strongly affects TG levels, but the potential clinical impact and underlying mechanisms are yet to be resolved. Here, we aimed to study the effects of *APOA5* genetic variation on CAD risk and plasma lipoproteins through factorial genetic association analyses. Using data from 309,780 European-ancestry participants from the UK Biobank, we evaluated the effects of lower TG levels as a result of genetic variation in *APOA5* and/or *LPL* on CAD risk with or without a background of reduced LDL-C. Next, we compared lower TG levels via *APOA5* and *LPL* variation with over 100 lipoprotein measurements in a combined sample from the Netherlands Epidemiology of Obesity study (N = 4,838) and the Oxford Biobank (N = 6,999). We found that lower TG levels due to combined *APOA5* and *LPL* variation and genetically-influenced lower LDL-C levels afforded the largest reduction in CAD risk (odds ratio: 0.78 (0.73–0.82)). Compared to patients with genetically-influenced lower TG via *LPL*, genetically-influenced lower TG via *APOA5* had similar and independent, but notably larger, effects on the lipoprotein profile. Our results suggest that lower TG levels as a result of *APOA5* variation have strong beneficial effects on CAD risk and the lipoprotein profile, which suggest apo A-V may be a potential novel therapeutic target for CAD prevention.

## Introduction

Current guidelines for coronary artery disease (CAD) prevention focus on statins as the first-line treatment aimed at reducing LDL-C. However, statins reduce cardiovascular risk by only approximately 20%–30% (1, 2). In addition to LDL-C, elevated levels of triglycerides (TG) and TG-rich lipoproteins (TRLs) have emerged as independent and causal risk factors for CAD (3, 4, 5).

Numerous genes have been linked to TG metabolism, among which *LPL*, which encodes LPL, has been shown to play a major role (6, 7). In addition to *LPL, APOA5*, encoding apolipoprotein A-V (apo A-V), is an important determinant of plasma TG levels (8, 9, 10). Apo A-V is mainly expressed in the liver and is present on and exchanged between TRLs and HDL-C (11, 12). Despite its low plasma concentration (≈150 ng/ml) compared with other apolipoproteins, apo A-V appears to be a potent regulator of circulating TG levels (13). In-vivo experiments found that mice overexpressing human *APOA5* had 66% lower plasma TG levels than controls, primarily due to a lower TG content in VLDL particles (14, 15). Reciprocally, *APOA5* knockout mice had a four-fold increase in plasma TG levels (14) and resembled apo A-V-deficient patients exhibiting type V familial hyperlipoproteinemia (10, 11). Furthermore, genome-wide association studies have identified rare and common variants in the *APOA5* locus to be associated with TG levels (8, 9, 16). Despite playing a crucial role in TG metabolism, the precise mechanism(s) through which apo A-V regulates TG levels remain under debate. Most evidence suggests that apo A-V enhances LPL-dependent TG lipolysis, either directly or indirectly (17, 18). Other hypotheses suggest that apo A-V regulates hepatic VLDL production (18) or facilitates the recognition of VLDL particles by members of the LDL receptor family and heparan sulfate proteoglycans (19, 20), thereby enhancing the clearance of these particles from the circulation.

Previously, factorial Mendelian Randomization analyses showed that genetically-influenced lower plasma TG levels via *LPL* have additional beneficial effects on reducing CAD risk on top of genetically-influenced lower LDL-C (21). As an important TG regulator, apo A-V could therefore be an interesting additional therapeutic target for CAD prevention. In the present study, we aimed to study *APOA5* genetic variation in relation to CAD, as well as the detailed lipoprotein profile, separately and in combination with variation in *LPL* and LDL-C-lowering through factorial genetic analyses in multiple cohorts.

## Materials and methods

### Study design and population

In this study, we aimed to: (1) assess the clinical relevance of genetically-influenced lower TG levels via *APOA5* and/or *LPL* variants on top of genetically-influenced lower LDL-C on CAD risk and (2) investigate the mechanisms of apo A-V relative to LPL by estimating the individual and combined associations with metabolomic measures of genetically-influenced lower TG via *APOA5* and genetically-influenced lower TG via *LPL*.

For the first aim, we performed single instrument and factorial genetic association analyses (supplemental Fig. S1–S3) using individual-level data from 309,780 CAD cases and controls in the UK Biobank. The UK Biobank cohort is a prospective general population cohort of 502,628 participants aged 40–70 years from across the United Kingdom. For the present study, we restricted the analyses to the UK Biobank participants who reported to be of European ethnicity, were unrelated (based on the availability of kinship data), and were present in the full release imputed genotyped datasets (N=309,780).

For the second aim, we used individual-level genetic data including 11,837 participants from a combined cross-sectional cohort of the Netherlands Epidemiology of Obesity (NEO) and the Oxford Biobank (OBB) study to perform genetic association analyses in 2 × 2 factorial design. The NEO study is a population-based prospective cohort study of 6,671 men and women aged 45–65 years. For the present study, we excluded participants with lipid-lowering drug use (n = 906) and/or missing data on genotype (n = 927). Therefore, the present study population consisted of 4,838 NEO participants. The OBB is a population-based cohort of 7,185 randomly selected healthy participants aged 30–50 years from Oxfordshire (UK). Individuals with a history of myocardial infarction, diabetes mellitus, heart failure, untreated malignancy, other ongoing systemic diseases, or ongoing pregnancy were not eligible for study inclusion. Participants with lipid-lowering drug use and missing genotype data were excluded, which resulted in a total of 6,999 participants included for the present study.

All included studies received ethical approval by their respective medical ethics committees (NEO was approved by the medical ethics committee of the Leiden University Medical Center, OBB was approved by the Oxfordshire Clinical Research Ethics Committee (08/H0606/107+5), and UK Biobank was approved by the North-West Multi-center Research Ethics Committee), and all participants gave their written informed consent. The studies conformed to the principles outlined in the Declaration of Helsinki. A more detailed description of the included studies, their designs, and the genotyping platforms is provided in supplementary methods and supplemental Table S1.

### Genetic instruments and genotype groups

In NEO, OBB, and UK Biobank, we calculated weighted genetic scores for both *APOA5* and *LPL* using TG-lowering alleles. For the *APOA5* genetic score, we used two variants (rs662799 and rs3135506; Extended Methods, supplemental Table S2) that comprise most of the variation in the *APOA5* locus, are in linkage equilibrium (*R*^2^=0.003), and are strongly associated with TG levels (22). Weights for the GRS calculation were derived from the genome-wide association study on TG levels from the Global Lipids Genetics Consortium (23). Likewise, the *LPL* genetic score was constructed using variants associated independently with TG levels that were mapped to the *LPL* gene (rs268, rs301, rs326, rs328, and rs10096633; Extended Methods, supplemental Table S2), which were weighted by their effect on TG levels in the analyses from Global Lipids Genetics Consortium (23). Based on the calculated GRS for *APOA5* and *LPL*, we divided the population based study on the median values of the two GRS resulting in 4 different study groups based on genetically-influenced apo A-V and LPL activity (2 × 2 factorial design, supplemental Fig. S2): (1) reference group (higher TG through *APOA5* and *LPL*), (2) lower TG through *LPL* only, (3) lower TG through *APOA*5 only, and (4) lower TG through both *APOA5* and *LPL*.

In UK Biobank, in addition to the *APOA5* and *LPL* genetic scores, we calculated a genetic LDL-C score by extracting from published genome-wide association studies in which the UK Biobank did not contribute the independent lead variants *(P* < 5 × 10^−8^) previously identified in relation to LDL-C levels (188,577 individuals; 15 SNPs, supplemental Table S2) (23). Using the beta estimates of the independent lead variants, we calculated weighted LDL-C genetic risk scores (GRS) per participant. To limit bias by pleiotropy, we did not allow overlap in independent lead variants between LDL-C and the other lipid traits (notably HDL-C and TG) based on a *p*-value cut-off of 5 × 10^−8^. Next, based on the weighted GRS of LDL-C, *LPL*, and *APOA5*, we stratified the study population into different groups based on the median values of the three GRS (supplemental Fig. S3).

### Study outcomes

#### Cardiovascular disease outcomes

In UK Biobank, the clinical outcome was CAD. Information on incident CAD was collected through information from the data provided by the NHS record systems. Diagnoses were coded according to the International Classification of Diseases (24). CAD was defined as: angina pectoris (I20), myocardial infarction (I21 and I22), and acute and chronic ischemic heart disease (I24 and I25).

#### NMR-based metabolomic profile

In NEO and OBB, the primary outcomes were the fasting NMR-based metabolomic measures. In both cohorts, a high-throughput proton NMR-metabolomics platform (25) (Nightingale Health Ltd., Helsinki, Finland) was used to measure 159 metabolic measures (excluding ratios) at the Medical Research Council Integrative Epidemiology Unit at the University of Bristol, Bristol, United Kingdom, which were quantified by Nightingale library. This method provides lipoprotein subclass profiling with lipid concentrations within 14 lipoprotein subclasses. Details of the experimentation and applications of the NMR-metabolomics platform have been described previously (25), as well as representative coefficients of variations for the metabolic biomarkers (26).

In this study, we excluded all ratios, resulting in a final number of 145 NMR-derived metabolic measures present in both NEO and OBB cohort. Values below the detection limit were treated as missing. For all analyses, metabolic measures were inverse rank transformed to obtain normal distributions.

### Statistical analyses

#### Factorial genetic association analyses with CAD risk in the UK Biobank cohort

We performed three types of genetic analyses on CAD cases and controls in the UK Biobank : 1, single instrument genetic analyses, where each dichotomized genetic score (LDL-C, *LPL*, and *APOA5* GRS) was associated with CAD outcomes, assuming that the other alleles were randomly distributed in the other groups (supplemental Fig. S1); 2, 2 × 2 factorial genetic analyses resulting from three different combinations (LDL-C-lowering and lower TG via *LPL* alleles, LDL-C-lowering and lower TG via *APOA5* alleles, and lower TG via both *LPL* and *APOA5* alleles) (supplemental Fig. S2); 3, 2 × 2 × 2 factorial genetic analyses with the combination of the three genetic scores to assess the clinical relevance of lower TG via *APOA5* and *LPL* variants on top of genetically-influenced lower LDL-C (supplemental Fig. S3).

Analyses in UK Biobank were performed in R (Version 3.6.1, the R Project, https://www.r-project.org/) using logistic regression adjusted for age, sex, and the first 10 principal components in unrelated individuals.

#### Factorial genetic association analyses with NMR-metabolomics

Using four “naturally randomized” subgroups based on *LPL* and *APOA5* GRS, we performed linear regression analyses to estimate the associations with NMR-based metabolomic measures between groups using a 2 × 2 factorial design in NEO and OBB separately. These association analyses were adjusted for age, sex, and the first four genomic principal components to correct for possible population stratification within the separate study samples. In addition, we included in the regression model an additive interaction term by using a product term between the continuous *LPL* and *APOA5* genetic scores to test whether they had additive effects on the NMR-based metabolomics measures. Finally, these analyses were also performed for replication purposes using nonfasting NMR-based metabolomics measures in the UK Biobank cohort.

All analyses in the NEO and OBB cohort were adjusted for multiple testing, dividing the alpha by 37, as this was the number of independent metabolic measures in our study. The number of independent biomarkers was determined using the method by Li and Ji (27). Associations were considered to be statistically significant in case the *p* value was below 1.35 × 10^−3^ (i.e., 0.05/37). All results for the NEO cohort were based on analyses weighted toward the reference BMI distribution of the general Dutch population and, therefore, apply to a population-based study without oversampling of individuals with overweight or obesity. A more detailed description of the weighting can be found elsewhere (28).

Finally, the separate results from the NEO and the OBB cohorts were meta-analyzed using the fixed-effect model of rmeta package in R. Linear regression analyses were carried out using STATA Statistical Software version 12.0 (Statacorp, College Station, Texas, USA) and R version 3.6.1 (The R Project, https://www.r-project.org/). The circular plots were designed using Python version 2.7.6 (Python Software Foundation, https://python.org/).

## Results

### Population characteristics

The UK Biobank study population investigated herein (Table 1) consisted of 309,780 participants (mean (SD): 56.8 (8.0) years of age at study inclusion), out of which 36,391 were CAD cases. Compared to the controls, the cases had a higher mean age (61.1 (6.4) vs. 56.2 (8.0) years, respectively) and a higher BMI (29.0 (5.0) and 27.2 (4.7) kg/m^2^ for cases and controls, respectively). In addition, the case group consisted of more male participants than the control group (66 vs. 43%, respectively).

**Table 1:** Characteristics of the UK Biobank total study population , as well as stratified in cases and controls

Characteristics	Total	Cases	Controls
Number of participants	309,780	36,391	273,389
Age at inclusion, years	56.8 (8.0)	61.1 (6.4)	56.2 (8.0)
Sex, % men	46	66	43
BMI (kg/m^2^)	27.4 (4.8)	29.0 (5.0)	27.2 (4.7)
Fasting serum concentrations (mmol/L)			
TG (median (IQR))	1.49 (1.1)	1.72 (1.25)	1.46 (1.08)
Total cholesterol	5.71 (1.14)	5.25 (1.28)	5.77 (1.11)
LDL-cholesterol	3.47(0.87)	3.27 (0.97)	3.61 (0.85)
HDL-cholesterol	1.45 (0.38)	1.30 (0.35)	1.47 (0.38)
LDL-C GRS (median (IQR))	0.41 (0.30)	0.39 (0.30)	0.41 (0.30)
a*LPL* GRS (median (IQR))	0.09 (0.20)	0.09 (0.20)	0.09 (0.23)
*APOA5* GRS (median (IQR))	0.86 (0.00)	0.86 (0.00)	0.86 (0.00)

In stratified analyses, the number of cases and controls varies per genotype group.

Values are mean (SD), unless otherwise specified. GRS unit is in SD.

GRS, genetic risk score; IQR, interquartile range.

a Due to unavailability of rs301, the *LPL* GRS for the UK Biobank was calculated based on five variants (rs268, rs326, rs328, and rs10096633) versus the six variants (rs268, rs301, rs326, rs328, and rs10096633) used in the NEO and OBB cohorts.

Characteristics of the NEO study population (N=4,838) and OBB cohort (N=6,999), as well as of the combined population are summarized in Table 2. Compared to participants from NEO, OBB participants had a lower mean age (41.6 (5.9) vs. 55.5 (6.0) years, respectively) but a similar mean BMI (25.8 (4.6) and 26.0 (4.3) kg/m^2^ for OBB and NEO, respectively). Levels of TG, total cholesterol, LDL-C, and HDL-C were higher in the NEO cohort than the OBB cohort.

**Table 2:** Characteristics of the NEO and the OBB cohort, as well as their combination

Characteristics	NEO^a^	OBB	Total^b^
Number of participants	4,838	6,999	11,837
Age (years)	55.5 (6.0)	41.6 (5.9)	47.3 (5.9)
Men (%)	42	44	43
BMI (kg/m^2^)	26.0 (4.3)	25.8 (4.6)	25.9 (4.5)
Fasting serum concentrations (mmol/L)			
TG (median (IQR))	0.99 (0.71)	0.93 (0.65)	0.95 (0.67)
Total cholesterol	5.80 (1.01)	5.18 (1.01)	5.43 (1.01)
LDL-cholesterol	3.66 (0.94)	3.22 (1.26)	3.40 (1.13)
HDL-cholesterol	1.60 (0.47)	1.38 (0.42)	1.47 (0.44)
*APOA5* GRS (median (IQR))	0.86 (0.00)	0.86 (0.00)	0.86 (0.00)
*LPL* GRS (median (IQR))	0.48 (0.24)	0.48 (0.24)	0.48(0.24)

Values are mean (SD), unless otherwise specified. GRS unit is in SD.

GRS, genetic risk score; IQR, interquartile range; NEO, Netherlands Epidemiology of Obesity; OBB, Oxford Biobank.

a In NEO, the results are based on analyses weighted toward the reference BMI distribution of the general Dutch population.

b The total represents averaged results from the individual analyses in NEO and OBB cohort.

### Factorial genetic association analyses with CAD risk

**Fig. 1:**
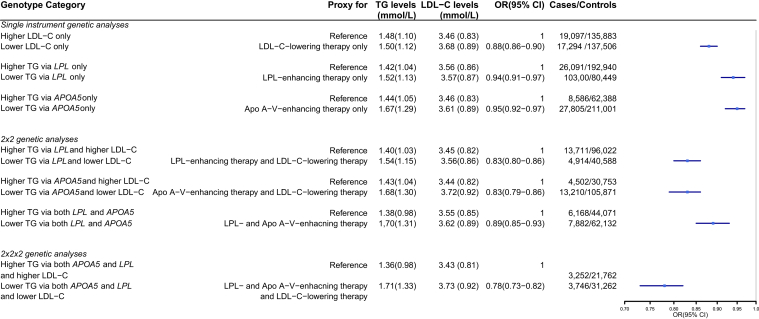
Associations of genotype group with Coronary Artery Disease in the UK Biobank cohort. Values are mean (SD) for LDL-C levels and median (IQR) for TG levels. GRS unit is in SD. CI, Confidence interval; OR, odds ratio; GRS, genetic risk scores.

The characteristics of the UK Biobank cohort stratified by genotype group based on the *LPL*, *APOA5*, and LDL-C GRS are shown in supplemental Table S3. Results from factorial genetic analyses with CAD in the UK Biobank are presented in Fig. 1. The group with lower TG via *APOA5* and groups with lower TG via *LPL* had a similar reduced odds ratio for CAD risk (OR (95% CI): 0.95 (0.92;0.97) vs. 0.94 (0.91;0.97), respectively). In addition, the effects of the genetic scores on CAD were also additive based on the comparison between the sum of the individual effects (*LPL*: OR=0.94; *APOA5*: OR=0.95) and the effect of both scores combined (both *LPL* and *APOA5*: OR=0.89). Based on an approximation of the OR with the risk ratio when the outcome incidence is <10%, the sum of the risk reduction of the individual *LPL* and *APOA5* scores translated into 9%, which was similar to the risk reduction in the group with both genetic exposures (11%). When combined with genetically-influenced lower LDL-C levels, genetically-influenced lower TG via *APOA5* were associated with the same CAD risk as the genetically lower TG via *LPL* (OR (95% CI):(0.83 (0.79;0.86) vs. 0.83 (0.80;0.86), respectively). The most beneficial effect on CAD risk was observed when genetically-influenced lower TG via both *LPL* and *APOA5* were combined with genetically-influenced lower LDL-C (OR (95% CI): (0.78 (0.73;0.82)).

### 2 × 2 factorial analyses with NMR-based metabolomic measures

**Fig. 2:**
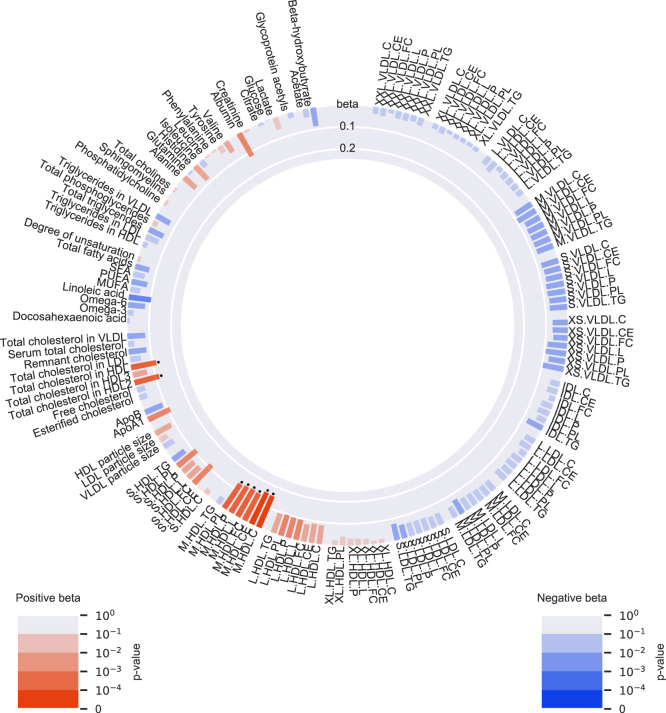
Associations of the group with genetically-influenced lower TG levels via *LPL* with 145 NMR-based metabolomic measures in 2 × 2 factorial analyses, in the Netherlands Epidemiology of Obesity (NEO) study (n = 4,838) and in the Oxford Biobank (OBB) cohort (n=6,999). Group with genetically-influenced lower TG levels via *LPL* compared with the reference group (genetically-influenced higher TG levels via both *LPL* and *APOA5*). Bar heights represent the magnitude of the beta coefficient from linear regression, which is expressed in SD units. Red bars indicate positive betas and blue bars indicate negative betas. The transparency of the bars indicates the level of statistical significance. A *p* <1.35 × 10^−3^ is regarded statistical significant, as represented by the black dots.

**Fig. 3:**
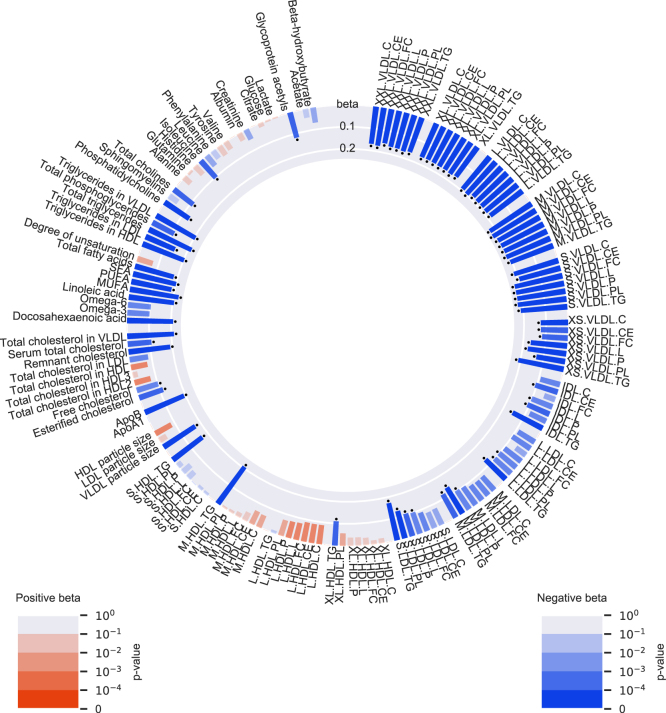
Associations of the group with genetically-influenced lower TG levels via *APOA5* with 145 NMR-based metabolomic measures in 2 × 2 factorial analyses, in the Netherlands Epidemiology of Obesity (NEO) study (n = 4,838) and in the Oxford Biobank (OBB) cohort (n=6,999). Group with genetically-influenced lower TG levels via *APOA5* compared with the reference group (genetically-influenced higher TG levels via both *LPL* and *APOA5*). Bar heights represent the magnitude of the beta coefficient from linear regression, which is expressed in SD units. Red bars indicate positive betas and blue bars indicate negative betas. The transparency of the bars indicates the level of statistical significance. A *p* <1.35 × 10^−3^ is regarded statistical significant, as represented by the black dots.

The characteristics of the combined population of NEO and OBB cohorts stratified by the dichotomized *LPL* and *APOA5* GRS are shown in supplemental Table S4. Compared with the reference group (genetically-influenced higher TG via both *LPL* and *APOA5)*, lower genetically-influenced TG levels via *LPL* only were associated with altered levels of eight metabolomic measures (particularly higher levels of medium-sized HDL subparticles; Figure 2 and supplemental Table S5) and lower genetically-influenced TG levels via *APOA5* only were associated with changed levels of 81 metabolomic measures (particularly lower levels of all sizes of VLDL subparticles; Figure 3 and supplemental Table S5). Despite these observed differences, in general, the effects of the *APOA5* and *LPL* genetic scores on the metabolomic measures showed a moderate overlap *R*^2^ = 0.68; supplemental Fig. S4).

**Fig. 4:**
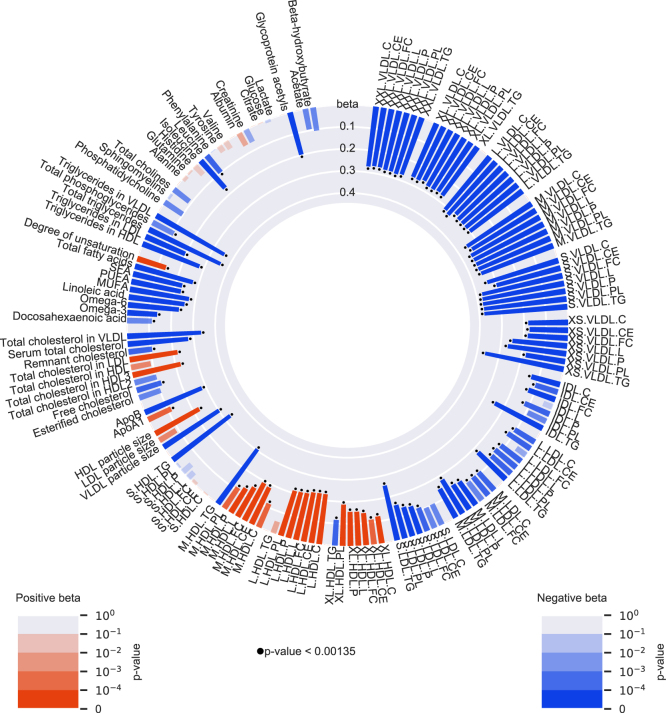
Associations of the group with genetically-influenced lower TG levels via both *LPL* and *APOA5* with 145 NMR-based metabolomic measures in 2 × 2 factorial analyses, in the Netherlands Epidemiology of Obesity (NEO) study (n = 4,838) and in the Oxford Biobank (OBB) cohort (n=6,999). Group with genetically-influenced lower TG levels via both *LPL* and *APOA5* compared with the reference group (genetically-influenced higher TG levels via both *LPL* and *APOA5*). Bar heights represent the magnitude of the beta coefficient from linear regression, which is expressed in SD units. Red bars indicate positive betas and blue bars indicate negative betas. The transparency of the bars indicates the level of statistical significance. A *p* <1.35 × 10^−3^ is regarded statistical significant, as represented by the black dots.

Compared to the same reference group, lower genetically-influenced TG levels via both *LPL* and *APOA5* were associated with altered levels of 86 metabolomic measures (Fig. 4 and supplemental Table S5). Overall, the effects of these associations showed an additive pattern of the individual associations of genetically-influenced lower TG levels via *APOA5* and genetically-influenced lower TG levels via *LPL* but no evidence for an interaction between these scores (*p* for interaction > 1.35 × 10^−3^). More specifically, the group with genetically-influenced lower TG levels via both *APOA5* and *LPL* was associated with lower levels of all VLDL subparticles and most LDL subparticles, as well as a lower average VLDL particle size (VLDLD: beta (SE) = −0.30 (0.03), *p* = 2.3 × 10^−23^). In line with these results, levels of apolipoprotein B (apoB), total serum cholesterol, cholesterol in VLDL (VLDL-C), and cholesterol in LDL (LDL-C) were also lower (apoB: beta (SE) = −0.28 (0.03), *p* = 3.6 × 10^−19^), whereas most HDL subparticles, HDL-C, and ApoA1 were higher (ApoA1: beta (SE) =0.12 (0.03), *p* = 2.2 × 10^−04^). In addition, genetically-influenced lower TG levels via both *LPL* and *APOA5* were associated with lower levels of total FAs (beta (SE) = −0.27 (0.06), *p* = 9.4 × 10^−17^) and several free FAs (omega-3, omega-6, monounsaturated FAs, polyunsaturated FAs, and short-chain FAs) and with a higher degree of unsaturation. Replication analyses in the UK Biobank cohort confirmed these observations, despite the fact that the metabolomics measurements were done irrespective of fasting status, which likely increased the variability of the measurements (supplemental Fig. S5).

## Discussion

In this study, exposure to genetically-influenced lower TG levels via *APOA5* had additional beneficial effects on CAD risk on top of genetically-influenced lower TG levels via *LPL* and genetically-influenced lower LDL-C levels. This was further supported by the independent and additive beneficial effects on the lipoprotein profile, of the genetically-influenced lower TG via *APOA5* on top of genetically-influenced lower TG via *LPL*. Therefore, our data suggests that pharmacological TG-lowering therapy via *APOA5* may have additional beneficial effects on the lipoprotein profile and CAD risk on top of LPL-enhancement therapy as well as LDL-C-lowering therapy.

Previously, it was reported that genetically-influenced lower TG levels through *LPL* have an additive lowering effect on CAD risk on top of genetically-influenced lower LDL-C (21, 29), which were confirmed by the beneficial effects of this combination on the lipoprotein profile recently shown by our group (30). The results from the current study extend these findings by suggesting that genetically-influenced lower TG via *APOA5* have similar beneficial effects on CAD risk and the lipoprotein profile as genetically-influenced lower TG via *LPL*. Collectively, genetically-influenced lower TG through *APOA5* and genetically-influenced lower TG through *LPL* were associated with an additively improved lipoprotein profile and CAD risk. More importantly, exposure to genetically-influenced lower TG levels via *APOA5* gave an additional reduction in primary CAD risk on top of exposure to genetically-influenced lower TG via *LPL* and genetically-influenced lower LDL-C levels. Data from other MR studies have shown that particularly, apoB may be the key trait accounting for the relationship between lipoproteins and CAD (29, 31). Since in our study both the *APOA5* and *LPL* genetic scores were associated with lower levels of VLDL subparticles and the LDL-C genetic score with lower levels of LDL subparticles, these all translated to lower levels of apoB. Thus, the observed reduction in CAD might be explained by lower levels of apoB, which was indeed the lowest in the group with the three genetic exposures. Altogether, these data suggest that apo A-V might be an attractive therapeutic target for additional treatment to reduce CAD risk. This opens up a novel avenue for the development of potentially effective drugs in CAD prevention, which is of high importance given the residual risk that remains in patients already on statin therapy (1, 2). One feasible approach, given the small size of the apo A-V (39 Kda), may be an *APOA5* expression construct targeted to muscle or liver.

Previously, association studies of *APOA5* variants with lipoprotein subparticles have been performed, although mostly with a less extensive metabolomics panel and limited cohort size. These studies showed the strongest associations of *APOA5* variants with chylomicrons and large VLDLs (32, 33, 34, 35), which is in line with the strong associations of lower TG via *APOA5* observed in our study. Guardiola *et al.* showed that the rare TG-increasing alleles the *APOA5* variants used in our study, notably rs3135506 and rs662799, were associated with an atherogenic lipoprotein profile (34). Similarly, in our study, we showed that the TG-lowering alleles of rs3135506 and rs662799 had a lowering effect on the atherogenic TRLs, including mostly VLDL subparticles. In addition, lower TG levels via *APOA5* were associated with lower levels of glycoprotein acetyls, a biomarker for inflammation (36), suggesting that *APOA5* may also play a role in atherogenesis by affecting inflammation. Sarwar *et al.* (33) reported no effect of *APOA5* on LDL, which is partially in concordance with our study, where we showed lower levels of only some of the LDL subparticles.

To our knowledge, the present study is the first showing the effects of lower TG via *APOA5* on an extensive NMR-metabolomic panel and its comparison with lower TG via *LPL*. Overall, the effect sizes of the associations of the *APOA5* alleles were stronger than those of the *LPL* alleles. Nevertheless, the directionality and pattern of these effects largely overlapped. In general, genetically-influenced lower TG levels via *APOA5* were predominantly associated with lower levels of VLDL subparticles and a smaller VLDL particle size and a lower number of particles, as indicated by apoB levels. Total cholesterol and total TG levels were lower in both, as well as total FAs. These associations could be due to enhanced TG hydrolysis, which is further confirmed by the higher levels of HLD subparticles and HDL particle size that result due to increased availability of surface components of TG-rich particles (37). However, these increasing effects on HDL subparticles were higher in the group with genetically-influenced lower TG via *LPL* than the group with genetically-influenced lower TG via *APOA5*. Except for the HDL subparticles, overall, the effect sizes of the associations with *APOA5* were larger than the effect sizes of the associations with *LPL*. Whether these effects are additional to LPL-dependent TG hydrolysis via other mechanisms, we cannot conclude based on the present findings. In addition to LPL-dependent TG hydrolysis, a role for apo A-V in hepatic VLDL production has been suggested by previous studies in mice (18). In addition to LPL-dependent TG hydrolysis and hepatic VLDL production, studies have shown that apo A-V also facilitates the recognition of TG-rich VLDL particles by the LDL receptor and heparan sulfate proteoglycans, thereby enhancing clearance of these particles (20). These potential other functions of apo A-V, we could not identify nor exclude with our present study design and need to be investigated in future studies. Nevertheless, from these results, we can conclude that *LPL* and *APOA5* are most likely associated with clinical outcomes via the same intermediates.

Several assumptions and limitations of the genetic approach used in this study should be considered when interpreting the results of our study. Mendelian randomization assumes that genetic variants are associated with the outcome only through the exposure of interest so that the results cannot be violated by (directional) pleiotropy. To take this assumption into account, we chose *APOA5* variants that are located within the *APOA5* gene: rs3135506 in the second exon and rs662799 located 2kb upstream of the *APOA5* gene. In addition, it has been previously found that rs3135506, also known as S19W, is a functional SNP that leads to an amino acid change, which subsequently leads to a 50% decrease in secretion, due to diminished translocation of apo A-V across the ER (38). Even though the effect of rs662799 on protein and functional level is less clear, rs662799 is in LD with rs2266788 (*R*^2^=0.77), which has been associated with *APOA5* gene expression (39). Although these data support our assumption that the observed effect on CAD via the *APOA5* genetic score occurs through apo A-V, we cannot formally exclude the possibility that alternative variants in linkage with variants in our *APOA5* GRS are the actual causative variants. Although the potential for such an alternative causative variant seems high given that *APOA5* is part of the *APOA1-C3-A4-A5* gene locus, such a variant remains to be identified. In addition, from the multitude of associations of the *APOA5* genetic score with the NMR profile (Fig. 3), we cannot conclude that the effect on CAD is mediated through the effect of apo A-V on plasma TG. As such, this analysis is not a proper Mendelian randomization analysis testing the causative effect of TG on CAD. Similarly, the *LPL* genetic score comprised variants that were in or within 10 kb of the *LPL* gene itself and were either coding variants associated with LPL function or significant expression quantitative trait loci (40, 41). This makes it likely that the genetically-influenced lower TG via the *LPL* genetic score truly resulted through LPL. But similar to *APOA5*, the *LPL* GRS is associated with a multitude of metabolites in the NMR profile (Fig. 2). Furthermore, we attempted to minimize possible pleiotropic effects of the LDL-C genetic score by including variants associated with LDL-C only, hence without associations to other lipid traits. Another potential limitation of our study is the inclusion of only two variants in the *APOA5* score, which in combination with a lower allele frequency could potentially lead to an underestimated effect estimate. Finally, our data are pertinent only to European populations, given that all the analyses in the NEO, OBB, and UK Biobank were performed in participants of European decent.

In summary, our study showed that genetically-influenced lower TG via *APOA5* have additional beneficial effects on CAD risk and lipoprotein profile, which were independent from and comparable to the effects of genetically-influenced lower TG via *LPL* alleles. Altogether, these results indicate that apo A-V is a potential novel therapeutic target for CAD prevention to be explored in detail in future studies.

## Data availability

Processed data for every figure described in the article are contained within the article and the supplementary materials. Because of consent issues, we cannot make the individual data of study participants available to other researchers for purposes of reproducing the results or replicating the procedure.

## Supplemental data

This article contains supplemental data (21, 22, 23, 24, 28, 38, 42, 43, 44, 45, 46, 47, 48).

## Conflict of interest

The authors declare that they have no conflicts of interest with the contents of this article.
