# Supplementary material for: Apolipoprotein A-V is a potential target for treating coronary artery disease: evidence from genetic and metabolomic analyses
Source: J Lipid Res. 2022 Mar 10;63(5):100193. doi: 10.1016/j.jlr.2022.100193 (PMC9062431; doi:10.1016/j.jlr.2022.100193)
Supplement: Supplementary material [file mmc1.pdf]

SUPPLEMENTAL MATERIAL

Apolipoprotein A-V is a potential therapeutic target for coronary artery disease: evidence from genetic and metabolomics analyses

Ibi. Apo-AV as a potential target for CAD prevention

Dorina Ibi[1,2*], Manon Boot[1], Martijn E.T. Dollé[2], J. Wouter Jukema[3,4] Frits R. Rosendaal[5], Constantinos Christodoulides[6], Matt J. Neville[6,7], Robert Koivula[6], Patrick C.N. Rensen[8,9], Fredrik Karpe[6,7], Raymond Noordam[10], Ko Willems van Dijk[1,8,9]

[1]Dept. Human Genetics, Leiden University Medical Center, Leiden, the Netherlands; [2]Dept. Public Health and Primary Care, National Institute for Public Health and the Environment (RIVM), Bilthoven, the Netherlands; [3]Dept. Cardiology, Leiden University Medical Center, Leiden, the Netherlands; [4]Netherlands Heart Institute, Utrecht, the Netherlands; [5]Dept. Clinical Epidemiology, Leiden University Medical Center, Leiden, the Netherlands; **[6]**Oxford Centre for Diabetes, Endocrinology and Metabolism, Radcliffe Department of Medicine, University of Oxford, Oxford, United Kingdom; [7]NIHR Oxford Biomedical Research Centre, Oxford University Hospitals Foundation Trust, Oxford, United Kingdom; [8]Dept. Internal Medicine, Div. Endocrinology, Leiden University Medical Center, Leiden, the Netherlands; [9]Einthoven Laboratory for Experimental Vascular Medicine, Leiden University Medical Center, Leiden, The Netherlands; [10]Dept. Internal Medicine, Div. Gerontology and Geriatrics, Leiden University Medical Center, Leiden, The Netherlands;;

[*] Corresponding author: Dorina Ibi MSc, Dept. Human Genetics, Leiden University Medical Center, Leiden, The Netherlands; Email d.ibi@lumc.nl

Extended Methods

1 Included studies and genotyping platforms

1.1 The Netherlands Epidemiology of Obesity Study

1.1.1 Study Population

This study is part of the Netherlands Epidemiology of Obesity (NEO) study, a population-based prospective cohort study of men and women aged 45 to 65 years. All inhabitants from the greater area of Leiden, The Netherlands, with a self-reported body mass index (BMI) of 27 kg/m^2 or higher were eligible to participate. Moreover, inhabitants from a nearby municipality (Leiderdorp, The Netherlands) in the same age group were asked to participate regardless of their BMI, thereby forming a population with a reference BMI distribution. Between September 2008 and September 2012, a total of 6,671 participants were included in the study.

Participants visited the NEO study centre for extensive physical examination. Research nurses used medication inventory to record current medication use. Prior to the study visit, participants completed questionnaires at home with respect to demographic, lifestyle, and clinical information. After an overnight fast of at least 10 hours, fasting blood samples were taken at the NEO study centre.

The NEO study was approved by the medical ethics committee of the Leiden University Medical Center (LUMC), and all participants gave their written informed consent. The study conformed to the principles outlined in the Declaration of Helsinki. Detailed information about the study design and data collection has been described elsewhere[1].

1.1.2 Genotyping and imputation

DNA was isolated from venous blood samples. Genotyping was performed using the Illumina HumanCoreExome-24 BeadChip (Illumina Inc., San Diego, California, United States of America). In the process of quality control, participants were excluded when 1) the sample call rate was <98%, 2) there was a sex mismatch, 3) heterozygosity rate was not within ±3 SD of mean heterozygosity rate, 4) participants

widely diverged based on the first two principal components (PCs) (±3.5 SD), 5) samples were duplicates, and 6) concordance with another DNA sample was >0.25 (related individuals). Genetic variants were excluded when 1) genotype call rate was <98%, and 2) variants were not in Hardy-Weinberg equilibrium (p-value $<1\times10^{-6}$). Detailed quality control steps have been described elsewhere[2]. Subsequently, genotypes were imputed to the 1000 Genome Project reference panel[3] (v3 2011) using IMPUTE (v2.2) software[4].

1.2 Oxford Biobank (OBB) Study

1.2.1 Study population

The OBB is a population-based cohort of randomly selected healthy participants aged 30 to 50 years from Oxfordshire (UK). Individuals with a history of myocardial infarction, diabetes mellitus, heart failure, untreated malignancy, other ongoing systemic diseases or ongoing pregnancy were not eligible for study inclusion. Participants were included between 1999 and May 2015. The OBB cohort comprises 7,185 individuals. A more detailed description of the study recruitment criteria and population characteristics is reported elsewhere[5].

1.2.2 Genotyping

For each OBB participant, 35 mL aliquots of whole blood were collected and frozen at -80°C for isolation of genomic DNA. Genotyping was performed using the Illumina Infinium Human Exome Beadchip 12v1 array platform for the first consecutive 5900 DNAs, and Affymetrix UK Biobank Axiom Array chip on the first consecutive 7500 participants[5]. In total 6,999 genotyped participants were included in the current study.

1.3 UK Biobank cohort

1.3.1 Study Population

The UK Biobank cohort is a prospective general population cohort. Baseline assessments took place between 2006 and 2010 in 22 different assessment centers across the United Kingdom[6]. A total of 502,628 participants between the age of 40 and 70 years were recruited from the general population. Invitation letters were sent to eligible adults registered to the National Health Services (NHS) and living within a 25 miles distance from one of the study assessment centers. The UK Biobank study was approved by the North-West Multi-center Research Ethics Committee (MREC). Access for information to invite participants was approved by the Patient Information Advisory Group (PIAG) from England and Wales. All participants in the UK Biobank study provided written informed consent. The project was completed under project number 56340.

1.3.2 Genotyping and genetic imputations in UK Biobank

UK Biobank genotyping was conducted by Affymetrix using a bespoke BiLEVE Axium array for approximately 50,000 participants; the remaining participants were genotyped using the Affymetrix UK Biobank Axiom array. All genetic data were quality controlled centrally by UK Biobank resources. More information on the genotyping processes can be found online (https://www.ukbiobank.ac.uk).

Based on the genotyped SNPs, UK Biobank resources performed centralized imputations on the autosomal SNPs using the UK10K haplotype[7], 1000 Genomes Phase 3 [3] and Haplotype Reference Consortium reference panels[8].

2 Selection of genetic variants

For this study, in both NEO and OBB we calculated two independent weighted *APOA5* and *LPL* genetic scores using TG-lowering alleles. Three common haplotypes comprise more than 95% of all variation of *APOA5* within the population and are defined by five different variants in the *APOA5* locus: rs3135506, rs662799, rs651821, rs2072560 and rs2266788 [9,10]. Since the last four variants are in full linkage disequilibrium, genotypes of rs3135506 and one of the four linkage disequilibrium variants are sufficient to define the three *APOA5* haplotypes. For this study we used the TG-lowering alleles of rs3135506

(rs3135506-G) and rs662799 (rs662799-A), which have been shown to be strongly associated with TG[10,11]. For each participant, we calculated weighted *APOA5* genetic TG score by summing the number of TG-lowering alleles for each variant, weighted by their effect on TG levels in the analyses of the Global Lipids Genetics Consortium (GLGC)[12]. Likewise, the *LPL* genetic score was constructed using variants associated with TG levels that were mapped to the *LPL* gene (rs268, rs301, rs326, rs328 and rs10096633), which were weighted by their effect on TG levels in the analyses of the GLGC[12]. All variants were independently and strongly associated with TG. More details on the selection of these *LPL* variants are described by Lotta et al[13]. In UK Biobank, in addition to the *APOA5* and *LPL* genetic scores calculated as described above, we extracted, from published genome-wide association studies in which the UK Biobank did not contribute, the independent lead variants (p-value<5×10^{-8}) previously identified in relation LDL-C levels (188,577 individuals; 15 SNPs)[12]. Using the beta estimates of the independent lead variants, we calculated weighted genetic risk scores per participant. To limit bias by pleiotropy, we did not allow overlap in independent lead variants between LDL-C and the other lipid traits.

References

1 de Mutsert R, den Heijer M, Rabelink TJ, Smit JWA, Romijn JA, Jukema JW, et al. The Netherlands Epidemiology of Obesity (NEO) study: study design and data collection. *Eur J Epidemiol*. 2013;28:513–23.

2 Blauw LL, Li-Gao R, Noordam R, de Mutsert R, Trompet S, Berbée JFP, et al. CETP (Cholesteryl Ester Transfer Protein) Concentration. *Circ Genomic Precis Med*. 2018;11.

3 1000 Genomes Project Consortium, Auton A, Brooks LD, Durbin RM, Garrison EP, Kang HM, et al. A global reference for human genetic variation. Nature. 2015;526:68-74.

4 Howie B, Fuchsberger C, Stephens M, Marchini J, Abecasis GR. Fast and accurate genotype imputation in genome-wide association studies through pre-phasing. *Nat Genet*. 2012;44:955–9.

5 Karpe F, Vasan SK, Humphreys SM, Miller J, Cheeseman J, Dennis AL, et al. Cohort profile: The Oxford Biobank. *Int J Epidemiol*. 2018;47:21-21g.

6 Sudlow C, Gallacher J, Allen N, Beral V, Burton P, Danesh J, et al. UK biobank: an open access resource for identifying the causes of a wide range of complex diseases of middle and old age. *PLoS Med*. 2015;12:e1001779.

7 UK10K Consortium, Walter K, Min JL, Huang J, Crooks L, Memari Y, et al. The UK10K project identifies rare variants in health and disease. *Nature*. 2015;526:82-90.

8 McCarthy S, Das S, Kretzschmar W, Delaneau O, Wood AR, Teumer A, et al. A reference panel of 64,976 haplotypes for genotype imputation. *Nat Genet*. 2016;48:1279-83.

9 Pennacchio LA, Olivier M, Hubacek JA, Krauss RM, Rubin EM, Cohen JC. Two independent apolipoprotein A5 haplotypes influence human plasma triglyceride levels. *Human molecular genetics*. 2002;11:3031-8.

10 Palmen J, Smith AJP, Dorfmeister B, Putt W, Humphries SE, Talmud PJ. The functional interaction on in vitro gene expression of APOA5 SNPs, defining haplotype APOA52, and their paradoxical

association with plasma triglyceride but not plasma apoAV levels. *Biochim Biophys Acta*. 2008;1782:447-452.

11 Talmud PJ, Palmen J, Putt W, Lins L, Humphries SE. Determination of the Functionality of Common APOA5 Polymorphisms. 2005;280(31):28215-20.

12 Global Lipids Genetics Consortium, Willer CJ, Schmidt EM, Sengupta S, Peloso GM, Gustafsson S, Kanoni S, et al. Discovery and Refinement of Loci Associated with Lipid Levels. Nat Genet. 2013;45(11):1–24.

13 Lotta LA, Stewart ID, Sharp SJ, Day FR, Burgess S, Luan J, et al. Association of Genetically Enhanced Lipoprotein Lipase–Mediated Lipolysis and Low-Density Lipoprotein Cholesterol–Lowering Alleles With Risk of Coronary Disease and Type 2 Diabetes. 2018;1–10.

Supplementary table 1. Included Studies and Genotyping Platforms

Study	Total No. Participants included	Genotyping Platform
NEO	4,838	Illumina HumanCoreExome-24 BeadChip
OBB	6,999	Illumina Infinium Human Exome Beadchip 12v1 & Affymetrix UK Biobank Axiom Array
UK Biobank	309,780	Affymetrix UK Biobank Axiom array

Abbreviations: NEO, Netherlands Epidemiology of Obesity; OBB, Oxford BioBank;

Supplementary table 2 List of five triglyceride-lowering *APOA5* and *LPL* variants and LDL-C lowering variants via 19 genetic genetic regions investigated in this study

Genetic score	SNP	Gene	EA[a]/NEA	EAF	Phenotype	Effect size (SD units)	SE	Reference[b]
Lower TG via *APOA5*	rs662799	*APOA5*	A/G	0.92	INT-triglycerides	-0.2474	0.0071	24097068
	rs3135506	*APOA5*	G/C	0.94	INT-triglycerides	-0.1837	0.0096	24097068
Lower TG via *LPL*	rs10096633	*LPL*	T/C	0.13	INT-triglycerides	-0.1471	0.0050	24097068
	rs301	*LPL*	C/T	0.24	INT-triglycerides	-0.1089	0.0039	24097068
	rs326	*LPL*	G/A	0.31	INT-triglycerides	- 0.0869	0.0050	24097068
	rs328	*LPL*	G/C	0.10	INT-triglycerides	-0.1670	0.0058	24097068
	rs268	*LPL*	A/G	0.98	INT-triglycerides	-0.1971	0.0364	30326043[c]
Lower LDL-C via 19 genetic regions								
	rs11136341	*PLEC1*	A/G	0.40	INT-LDL-C	-0.045	0.0066	24097068
	rs11563251	*UGT1A1*	C/T	0.12	INT-LDL-C	-0.034	0.0062	24097068
	rs12027135	*LDLRAP1*	A/T	0.46	INT-LDL-C	-0.03	0.0039	24097068
	rs12916	*HMGCR*	T/C	0.40	INT-LDL-C	-0.073	0.0039	24097068
	rs2030746	*LOC84931*	C/T	0.40	INT-LDL-C	-0.021	0.0037	24097068
	rs2072183	*NPC1L1*	G/C	0.29	INT-LDL-C	-0.039	0.0048	24097068
	rs2328223	*SNX5*	A/C	0.21	INT-LDL-C	-0.03	0.0052	24097068
	rs2642442	*MOSC1*	C/T	0.33	INT-LDL-C	-0.036	0.0055	24097068
	rs2710642	*EHBP1*	G/A	0.35	INT-LDL-C	-0.024	0.0041	24097068
	rs2902940	*MAFB*	G/A	0.30	INT-LDL-C	-0.027	0.0040	24097068
	rs2954029	*TRIB1*	T/A	0.47	INT-LDL-C	-0.056	0.0037	24097068
	rs4299376	*ABCG5*	T/G	0.31	INT-LDL-C	-0.081	0.0045	24097068
	rs4942486	*BRCA2*	C/T	0.48	INT-LDL-C	-0.024	0.0036	24097068
	rs514230	*IRF2BP2*	A/T	0.48	INT-LDL-C	-0.036	0.0053	24097068
	rs6029526	*TOP1*	T/A	0.47	INT-LDL-C	-0.044	0.0051	24097068
	rs7206971	*OSBPL7*	G/A	0.49	INT-LDL-C	-0.029	0.0057	24097068
	rs9488822	*FRK*	A/T	0.36	INT-LDL-C	-0.031	0.0060	24097068
	rs964184	*APOA1*	C/G	0.84	INT-LDL-C	-0.086	0.0081	24097068
	rs9987289	*PPP1R3B*	A/G	0.10	INT-LDL-C	-0.071	0.0071	24097068

Abbreviations: EA, effect allele; NEA, non-effect allele; EAF, effect allele frequency; SNP, single nucleotide polymorphism; SE, standard error; *LPL, lipoprotein lipase*; LDL-C, low-density lipoprotein cholesterol; *PLEC1, Plectin; UGT1A, UDP Glucuronosyltransferase Family 1 Member A; LDLRAP, Low Density Lipoprotein Receptor Adaptor Protein 1 HMGCR, 3-Hydroxy-3-Methylglutaryl-CoA Reductase; NPC1L1, Niemann-Pick*

C1-Like 1; PCSK9, Proprotein convertase subtilisin/kexin type 9; SNX5, Sorting Nexin 5; MOSC1, Molybdenum Cofactor Sulfurase C-terminal Domain-Containing; EHBP1, EH Domain Binding Protein 1; MAFB, MAF BZIP Transcription Factor B; TRIB1, Tribbles Pseudokinase 1; ABCG5, ATP Binding Cassette Subfamily G Member 5; BRCA2, Breast Cancer Type 2 Susceptibility Protein; IRF2BP2, Interferon Regulatory Factor 2 Binding Protein 2; TOP1, DNA Topoisomerase I; OSBPL7, Oxysterol Binding Protein Like 7; FRK, Fyn Related Src Family Tyrosine Kinase; APOA1, Apolipoprotein A1; PPP1R3B, Protein Phosphatase 1 Regulatory Subunit 3B.

a The effect allele is the triglyceride-lowering allele or the lipid-lowering allele.

b PubMed ID of the original manuscript from which beta coefficients and standard errors are derived.

c Estimated in EPIC-Norfolk

Supplementary table 3 Characteristics of the UK BioBank study stratified by the LDL-C, *LPL* and *APOA5* genetic risk scores

Characteristics	LDL-C-lowering		TG-lowering via *LPL*		TG-lowering via *APOA5*		LDL-C lowering + TG-lowering via *LPL*		LDL-C-lowering + TG-lowering via *APOA5*		TG-lowering via *LPL* and *APOA5*		LDL-C-lowering + TG-lowering via *LPL* and *APOA5*	
	Yes	No	Yes	No	Yes	No	Yes	No	Yes	No	Yes	No	Yes	No
N	154,800	154,980	90,749	219,031	238,806	70,974	45,502	109,733	119,081	35,255	7,0014	50,239	35,008	25,014
LDL-C GRS (median (IQR))	0.56 (0.19)	0.26 (0.19)	0.41 (0.30)	0.41 (0.30)	0.41 (0.30)	0.41 (0.30)	0.56 (0.19)	0.26 (0.19)	0.56 (0.19)	0.26 (0.19)	0.41 (0.30)	0.41 (0.30)	0.56 (0.19)	0.26 (0.19)
LPL GRS (median (IQR))	0.09 (0.23)	0.09 (0.20)	0.40 (0.17)	0.00 (0.09)	0.09 (0.23)	0.09 (0.20)	0.40 (0.17)	0.00 (0.09)	0.09 (0.23)	0.09 (0.20)	0.40 (0.17)	0.00 (0.09)	0.40 (0.17)	0.00 (0.09)
APOA5 GRS (median (IQR))	0.86 (0.00)	0.86 (0.00)	0.86 (0.00)	0.86 (0.00)	0.86 (0.00)	0.61 (0.06)	0.86 (0.00)	0.86 (0.00)	0.86 (0.00)	0.61 (0.06)	0.86 (0.00)	0.61 (0.06)	0.86 (0.00)	0.61 (0.06)
Age, years	56.8 (8.0)	56.7 (8.0)	56.8 (8.0)	56.7 (8.0)	56.8 (8.0)	56.8 (8.0)	56.8 (8.0)	56.7 (8.0)	56.8 (8.0)	56.7 (8.0)	56.8 (8.0)	56.7 (8.0)	56.8 (8.0)	56.7 (8.0)
Sex, men	46	46	46	46	46	46	46	46	46	45	46	45	46	45
Body mass index, kg/m2	27.4 (4.8)	27.4 (4.8)	27.3 (4.7)	27.4 (4.8)	27.4 (4.8)	27.4 (4.8)	27.4 (4.7)	27.4 (4.8)	27.4 (4.8)	27.4 (4.8)	27.3 (4.7)	27.4 (4.8)	27.4 (4.8)	27.4 (4.8)
Fasting serum concentrations (mmol/L):														
TG (median (IQR))	1.48 (1.10)	1.50 (1.12)	1.42 (1.04)	1.52 (1.13)	1.44 (1.05)	1.67 (1.29)	1.40 (1.03)	1.54 (1.15)	1.43 (1.04)	1.68 (1.30)	1.38 (0.98)	1.70 (1.31)	1.36 (0.98)	1.71 (1.33)
Total cholesterol	5.59 (1.09)	5.84 (1.17)	5.71 (1.13)	5.71 (1.15)	5.69 (1.13)	5.78 (1.18)	5.59 (1.08)	5.84 (1.18)	5.56 (1.08)	5.91 (1.21)	5.69 (1.11)	5.78 (1.18)	5.56 (1.06)	5.91 (1.22)
LDL cholesterol	3.46 (0.83)	3.68 (0.89)	3.56 (0.86)	3.57 (0.87)	3.46 (0.83)	3.61 (0.89)	3.45 (0.82)	3.56 (0.86)	3.44 (0.82)	3.72 (0.92)	3.55 (0.85)	3.62 (0.89)	3.43 (0.81)	3.73 (0.92)
HDL cholesterol	1.46 (0.39)	1.45 (0.38)	1.48 (0.39)	1.44 (0.38)	1.46 (0.39)	1.42 (0.38)	1.49 (0.39)	1.46 (0.38)	1.47 (0.39)	1.41 (0.37)	1.49 (0.39)	1.41 (0.37)	1.50 (0.39)	1.40 (0.37)

Values are mean (SD), unless otherwise specified. GRS unit is in SD. [a]Due to nonavailability of rs301, the *LPL* GRS for the UK BioBank was calculated based on five variants (rs268, rs326, rs328 and rs10096633) versus the six variants (rs268, rs301, rs326, rs328 and rs10096633) used in the NEO and OBB cohorts.
Abbreviations: BMI, body mass index; GRS, genetic risk score; HDL, high-density lipoprotein; IQR, interquartile range; LDL, low-density lipoprotein; TG, triglycerides

Supplementary table 4 Characteristics of the combined cohort of the Netherlands Epidemiology of Obesity (NEO) study and the Oxford Biobank (OBB) study stratified by the *LPL* and *APOA5* genetic risk scores

**Characteristics**	**1: Reference**	**2: TG-lowering via *LPL***	**3: TG-lowering via *APOA5***	**4: TG-lowering via both**
Total (n)	1,908	1,657	4,547	3,725
Age (years)	49.0 (6.0)	48.8 (6.0)	46.5 (5.8)	46.7 (5.9)
Men (%)	41.8	40.3	44.3	43.5
BMI (kg/m^2)	26.1 (4.5)	25.9 (4.5)	25.9 (4.4)	25.8 (4.5)
Fasting serum concentrations (mmol/L):				
Total cholesterol	5.61 (1.01)	5.55 (1.03)	5.37 (0.98)	5.36 (0.97)
LDL-cholesterol	3.57 (0.91)	3.49 (0.94)	3.41 (0.87)	3.38 (0.88)
HDL-cholesterol	1.44 (0.41)	1.52 (0.43)	1.44 (0.40)	1.50 (0.42)
Triglycerides (median (IQR)	1.05 (0.77; 1.58)	1.02 (0.72; 1.47)	0.96 (0.70; 1.37)	0.89 (0.66; 1.26)
LPL GRS (median (IQR)	0.39 (0.00)	0.72 (0.31)	0.39 (0.00)	0.72 (0.31)
APOA5 GRS (median (IQR)	0.64 (0.14)	0.64 (0.14)	0.86 (0.00)	0.86 (0.00)

Values are mean (SD), unless otherwise specified. Genetic risk score unit is in SD.
Data represent averaged results from the individual analyses in NEO and OBB cohort
In NEO, results are based on analyses weighted towards the reference BMI distribution of the general Dutch population.
Abbreviations: BMI, body mass index; GRS, genetic risk score; HDL, high-density lipoprotein; IQR, interquartile range; LDL, low-density lipoprotein; TG, triglycerides

Supplementary table 5 The associations between genetically-influenced lower triglyceride levels via *APOA5* and *LPL*, separately and in combination and 145 NMR-based metabolomic measures in the combined cohort of Netherlands Epidemiology of Obesity (NEO) study (n = 4,838) and the OBB study (n= 6,999)

	**Lower TG via *LPL***			**Lower LDL-C**			**Lower TG via *LPL* & *APOA5***		
**Metabolic measure**	**BETA***	**SE**	***P* VALUE**	**BETA**	**SE**	***P* VALUE**	**BETA***	**SE**	***P* VALUE**
Very-low-density lipoproteins (VLDL)									
XXL.VLDL.C	-0.161	0.065	1.41E-02	-0.106	0.060	7.58E-02	-0.316	0.062	3.25E-07
XXL.VLDL.CE	-0.141	0.064	2.70E-02	-0.109	0.059	6.41E-02	-0.285	0.060	2.28E-06
XXL.VLDL.FC	-0.156	0.066	1.77E-02	-0.082	0.060	1.70E-01	-0.313	0.062	4.29E-07
XXL.VLDL.L	-0.163	0.066	1.40E-02	-0.096	0.060	1.06E-01	-0.326	0.062	1.55E-07
XXL.VLDL.P	-0.163	0.066	1.39E-02	-0.096	0.059	1.05E-01	-0.327	0.062	1.44E-07
XXL.VLDL.PL	-0.170	0.067	1.15E-02	-0.099	0.059	9.62E-02	-0.327	0.062	1.16E-07
XXL.VLDL.TG	-0.162	0.066	1.44E-02	-0.094	0.059	1.41E-01	-0.326	0.062	1.44E-07
XL.VLDL.C	-0.131	0.064	4.01E-02	-0.117	0.060	5.00E-02	-0.284	0.058	1.26E-06
XL.VLDL.CE	-0.136	0.063	3.20E-02	-0.114	0.059	5.64E-02	-0.279	0.058	1.52E-06
XL.VLDL.FC	-0.120	0.065	6.30E-02	-0.115	0.060	5.75E-02	-0.289	0.060	1.40E-06
XL.VLDL.L	-0.120	0.066	6.93E-02	-0.086	0.062	1.67E-01	-0.289	0.062	2.95E-06
XL.VLDL.P	-0.118	0.066	7.39E-02	-0.082	0.062	1.85E-01	-0.287	0.062	3.49E-06
XL.VLDL.PL	-0.122	0.066	6.47E-02	-0.106	0.062	8.73E-02	-0.303	0.062	9.45E-07
XL.VLDL.TG	-0.115	0.066	8.11E-02	-0.074	0.062	2.36E-01	-0.284	0.062	4.67E-06
L.VLDL.C	-0.132	0.057	2.04E-02	-0.109	0.055	4.57E-02	-0.321	0.056	1.20E-08
L.VLDL.CE	-0.135	0.056	1.61E-02	-0.112	0.054	3.91E-02	-0.331	0.056	4.31E-09
L.VLDL.FC	-0.125	0.058	3.22E-02	-0.102	0.056	6.89E-02	-0.288	0.056	2.43E-07
L.VLDL.L	-0.133	0.059	2.36E-02	-0.102	0.056	7.10E-02	-0.304	0.057	8.01E-08
L.VLDL.P	-0.133	0.059	2.45E-02	-0.100	0.056	7.63E-02	-0.303	0.057	9.43E-08
L.VLDL.PL	-0.134	0.059	2.41E-02	-0.105	0.056	6.19E-02	-0.308	0.057	6.18E-08
L.VLDL.TG	-0.130	0.059	2.79E-02	-0.095	0.057	9.44E-02	-0.295	0.057	2.08E-07
M.VLDL.C	-0.189	0.057	9.32E-04	-0.157	0.054	3.73E-03	-0.380	0.054	3.55E-12
M.VLDL.CE	-0.181	0.057	1.56E-03	-0.163	0.054	2.50E-03	-0.378	0.055	5.34E-12
M.VLDL.FC	-0.189	0.057	9.11E-04	-0.146	0.054	7.54E-03	-0.364	0.054	2.22E-11
M.VLDL.L	-0.194	0.057	7.10E-04	-0.143	0.054	8.29E-03	-0.374	0.055	1.11E-11

M.VLDL.P	-0.194	0.057	7.06E-04	-0.142	0.054	8.97E-03	-0.372	0.055	1.36E-11
M.VLDL.PL	-0.193	0.057	7.56E-04	-0.149	0.054	6.27E-03	-0.379	0.055	6.85E-12
M.VLDL.TG	-0.190	0.057	8.42E-04	-0.131	0.054	1.61E-02	-0.361	0.055	5.75E-11
S.VLDL.C	-0.195	0.061	1.55E-03	-0.184	0.058	1.38E-03	-0.424	0.061	4.41E-12
S.VLDL.CE	-0.182	0.062	3.12E-03	-0.188	0.058	1.33E-03	-0.405	0.062	6.95E-11
S.VLDL.FC	-0.203	0.060	7.91E-04	-0.173	0.057	2.24E-03	-0.412	0.058	1.90E-12
S.VLDL.L	-0.212	0.060	4.17E-04	-0.165	0.056	3.36E-03	-0.415	0.058	1.25E-12
S.VLDL.P	-0.212	0.060	3.88E-04	-0.161	0.056	4.22E-03	-0.410	0.058	1.72E-12
S.VLDL.PL	-0.206	0.061	7.88E-04	-0.169	0.057	3.15E-03	-0.409	0.059	4.27E-12
S.VLDL.TG	-0.201	0.058	5.11E-04	-0.133	0.055	1.60E-02	-0.370	0.056	4.64E-11
XS.VLDL.C	-0.111	0.059	6.11E-02	-0.161	0.057	4.46E-03	-0.315	0.061	2.26E-07
XS.VLDL.CE	-0.108	0.060	6.89E-02	-0.149	0.057	8.87E-03	-0.301	0.061	7.84E-07
XS.VLDL.FC	-0.112	0.059	5.80E-02	-0.175	0.056	1.66E-03	-0.326	0.060	6.24E-08
XS.VLDL.L	-0.145	0.060	1.56E-02	-0.178	0.057	1.68E-03	-0.372	0.060	6.92E-10
XS.VLDL.P	-0.154	0.060	1.06E-02	-0.179	0.057	1.69E-03	-0.384	0.060	1.77E-10
XS.VLDL.PL	-0.104	0.058	7.00E-02	-0.180	0.054	9.20E-04	-0.321	0.058	3.25E-08
XS.VLDL.TG	-0.199	0.060	9.14E-04	-0.139	0.057	1.52E-02	-0.373	0.058	1.21E-10
Intermediate-density (IDL)	*lipoproteins*								
IDL.C	-0.044	0.056	4.33E-01	-0.163	0.053	2.07E-03	-0.251	0.058	1.38E-05
IDL.CE	-0.056	0.057	3.28E-01	-0.168	0.054	1.85E-03	-0.274	0.059	3.17E-06
IDL.FC	-0.016	0.054	7.61E-01	-0.150	0.051	3.26E-03	-0.189	0.055	5.55E-04
IDL.L	-0.051	0.056	3.60E-01	-0.163	0.053	1.98E-03	-0.259	0.057	5.52E-06
IDL.P	-0.057	0.056	3.10E-01	-0.164	0.053	1.88E-03	-0.269	0.057	2.56E-06
IDL.PL	-0.043	0.055	4.28E-01	-0.163	0.052	1.70E-03	-0.238	0.056	2.02E-05
IDL.TG	-0.120	0.058	3.97E-02	-0.125	0.057	2.80E-02	-0.285	0.057	6.20E-07
Low-density lipoproteins (LDL)									
L.LDL.C	-0.043	0.056	4.44E-01	-0.170	0.052	1.21E-03	-0.255	0.057	9.07E-06
L.LDL.CE	-0.052	0.057	3.60E-01	-0.174	0.053	9.93E-04	-0.273	0.058	2.61E-06
L.LDL.FC	-0.017	0.054	7.56E-01	-0.156	0.051	2.20E-03	-0.199	0.055	2.94E-04
L.LDL.L	-0.045	0.056	4.18E-01	-0.170	0.052	1.15E-03	-0.261	0.057	4.94E-06
L.LDL.P	-0.049	0.056	3.86E-01	-0.172	0.053	1.10E-03	-0.267	0.057	3.03E-06

L.LDL.PL	-0.047	0.056	4.01E-01	-0.175	0.052	8.08E-04	-0.267	0.057	2.80E-06
L.LDL.TG	-0.057	0.056	3.05E-01	-0.122	0.054	2.44E-02	-0.218	0.055	7.75E-05
M.LDL.C	-0.042	0.057	4.63E-01	-0.167	0.053	1.65E-03	-0.247	0.057	1.83E-05
M.LDL.CE	-0.046	0.057	4.20E-01	-0.168	0.053	1.56E-03	-0.250	0.058	1.47E-05
M.LDL.FC	-0.026	0.057	6.46E-01	-0.162	0.053	2.21E-03	-0.235	0.057	4.52E-05
M.LDL.L	-0.047	0.057	4.12E-01	-0.171	0.053	1.34E-03	-0.261	0.057	5.58E-06
M.LDL.P	-0.049	0.057	3.91E-01	-0.171	0.053	1.30E-03	-0.264	0.057	4.21E-06
M.LDL.PL	-0.065	0.059	2.65E-01	-0.185	0.054	6.37E-04	-0.308	0.058	1.15E-07
M.LDL.TG	-0.031	0.055	5.70E-01	-0.119	0.054	2.71E-02	-0.176	0.055	1.37E-03
S.LDL.C	-0.032	0.057	5.76E-01	-0.162	0.053	2.22E-03	-0.231	0.057	5.63E-05
S.LDL.CE	-0.037	0.057	5.20E-01	-0.163	0.053	1.95E-03	-0.232	0.057	4.73E-05
S.LDL.FC	-0.021	0.057	7.18E-01	-0.156	0.054	3.66E-03	-0.226	0.058	1.10E-04
S.LDL.L	-0.039	0.058	4.96E-01	-0.168	0.053	1.63E-03	-0.253	0.058	1.17E-05
S.LDL.P	-0.043	0.058	4.59E-01	-0.170	0.053	1.48E-03	-0.260	0.058	6.94E-06
S.LDL.PL	-0.039	0.058	5.01E-01	-0.173	0.054	1.36E-03	-0.270	0.058	3.32E-06
S.LDL.TG	-0.101	0.057	7.75E-02	-0.149	0.055	6.91E-03	-0.287	0.055	1.99E-07
High-density lipoproteins (HDL)									
XL.HDL.C	0.127	0.054	1.88E-02	-0.027	0.052	6.06E-01	0.126	0.054	1.98E-02
XL.HDL.CE	0.123	0.054	2.40E-02	-0.034	0.052	5.15E-01	0.115	0.055	1.58E-02
XL.HDL.FC	0.138	0.053	9.45E-03	-0.007	0.051	8.93E-01	0.153	0.054	4.57E-03
XL.HDL.L	0.132	0.052	1.09E-02	-0.008	0.050	8.80E-01	0.157	0.053	2.91E-03
XL.HDL.P	0.131	0.052	1.14E-02	-0.007	0.050	8.81E-01	0.157	0.053	2.93E-03
XL.HDL.PL	0.138	0.052	7.74E-03	-0.014	0.050	7.84E-01	0.196	0.052	1.88E-04
XL.HDL.TG	0.015	0.059	8.01E-01	-0.061	0.054	2.55E-01	-0.081	0.056	1.49E-01
L.HDL.C	0.176	0.054	1.13E-03	0.069	0.051	1.82E-01	0.235	0.054	1.63E-05
L.HDL.CE	0.176	0.054	1.08E-03	0.070	0.052	1.74E-01	0.236	0.054	1.54E-05
L.HDL.FC	0.175	0.054	1.20E-03	0.066	0.051	2.02E-01	0.232	0.054	2.00E-05
L.HDL.L	0.169	0.053	1.39E-03	0.057	0.051	2.61E-01	0.216	0.054	5.54E-05
L.HDL.P	0.168	0.053	1.47E-03	0.055	0.051	2.76E-01	0.213	0.054	6.83E-05
L.HDL.PL	0.163	0.053	1.88E-03	0.048	0.051	3.41E-01	0.203	0.053	1.47E-04
L.HDL.TG	0.104	0.055	5.69E-02	0.005	0.051	9.18E-01	0.093	0.055	9.16E-02
M.HDL.C	0.140	0.053	7.58E-03	0.011	0.050	8.27E-01	0.145	0.051	4.32E-03

M.HDL.CE	0.144	0.053	6.68E-03	0.016	0.050	7.54E-01	0.151	0.051	3.23E-03
M.HDL.FC	0.126	0.052	1.44E-02	-0.007	0.049	8.81E-01	0.123	0.050	1.44E-02
M.HDL.L	0.117	0.052	2.50E-02	-0.010	0.050	8.38E-01	0.101	0.051	4.78E-02
M.HDL.P	0.112	0.052	3.25E-02	-0.014	0.050	7.77E-01	0.090	0.051	7.76E-02
M.HDL.PL	0.110	0.052	3.34E-02	-0.017	0.050	7.30E-01	0.092	0.051	7.19E-02
M.HDL.TG	-0.165	0.061	6.71E-03	-0.128	0.057	2.43E-02	-0.309	0.058	1.05E-07
S.HDL.C	0.044	0.059	4.54E-01	-0.067	0.053	2.06E-01	-0.049	0.056	3.81E-01
S.HDL.CE	0.033	0.058	5.68E-01	-0.079	0.053	1.34E-01	-0.070	0.055	2.09E-01
S.HDL.FC	0.063	0.060	2.93E-01	0.011	0.056	8.42E-01	0.044	0.057	4.44E-01
S.HDL.L	0.027	0.063	6.64E-01	-0.040	0.057	4.89E-01	-0.048	0.061	4.32E-01
S.HDL.P	0.017	0.063	7.86E-01	-0.044	0.058	4.50E-01	-0.062	0.061	3.13E-01
S.HDL.PL	0.032	0.061	5.98E-01	0.018	0.056	7.55E-01	0.017	0.059	7.75E-01
S.HDL.TG	-0.189	0.059	1.48E-03	-0.100	0.057	8.00E-02	-0.298	0.057	2.15E-07
Lipoprotein particle size									
VLDL particle size	-0.165	0.054	2.12E-03	-0.082	0.052	1.15E-01	-0.266	0.052	3.83E-07
LDL particle size	-0.009	0.061	8.78E-01	0.043	0.053	4.12E-01	0.050	0.058	3.84E-01
HDL particle size	0.164	0.061	1.95E-03	0.027	0.051	5.90E-01	0.221	0.053	3.26E-05
Apolipoproteins									
ApoA1	0.139	0.054	5.54E-03	-0.041	0.049	3.97E-01	0.068	0.051	1.85E-01
ApoB	-0.133	0.068	2.37E-02	-0.185	0.055	8.07E-01	-0.387	0.058	3.64E-11
Cholesterols									
Esterified cholesterol	-0.003	0.055	9.58E-01	-0.162	0.052	1.77E-03	-0.219	0.056	9.02E-05
Free cholesterol	-0.017	0.054	7.61E-01	-0.146	0.052	5.00E-03	-0.195	0.056	4.91E-04
Total cholesterol in HDL2	0.170	0.050	6.13E-04	0.014	0.049	7.71E-01	0.198	0.050	6.70E-05
Total cholesterol in HDL3	0.113	0.051	2.67E-02	-0.058	0.050	2.48E-01	0.052	0.051	3.09E-01
Total cholesterol in HDL	0.174	0.050	4.89E-04	0.010	0.049	8.30E-01	0.195	0.050	8.66E-05
Total cholesterol in LDL	-0.038	0.056	4.99E-01	-0.166	0.053	1.57E-03	-0.250	0.057	1.34E-05
Remnant cholesterol	-0.135	0.059	2.18E-02	-0.178	0.055	1.26E-03	-0.380	0.059	9.15E-11
Serum total cholesterol	-0.007	0.055	8.94E-01	-0.158	0.052	2.23E-03	-0.216	0.056	1.07E-04

Total cholesterol in VLDL	-0.184	0.059	1.80E-03	-0.163	0.055	3.11E-03	-0.407	0.058	2.43E-12
Fatty acids									
Conjugated linoleic acid	0.012	0.069	8.61E-01	-0.090	0.064	1.61E-01	-0.070	0.066	2.86E-01
Docosahexaenoic acid	-0.030	0.062	6.30E-01	-0.044	0.060	4.65E-01	-0.111	0.062	7.40E-02
Fatty acid chain length	-0.045	0.066	4.99E-01	0.005	0.062	9.34E-01	0.049	0.064	4.41E-01
Omega-3	-0.035	0.058	5.53E-01	-0.089	0.057	1.15E-01	-0.210	0.060	4.51E-04
Omega-6	-0.077	0.058	1.84E-01	-0.174	0.055	1.60E-03	-0.275	0.059	2.97E-06
Linoleic acid	-0.103	0.055	6.27E-02	-0.215	0.054	7.79E-05	-0.327	0.057	8.71E-09
MUFA	-0.077	0.059	1.90E-01	-0.096	0.055	7.88E-02	-0.244	0.056	1.27E-05
PUFA	-0.074	0.058	1.99E-01	-0.166	0.055	2.60E-03	-0.279	0.059	2.56E-06
SFA	-0.055	0.059	3.50E-01	-0.076	0.055	1.69E-01	-0.253	0.059	1.56E-05
Total fatty acids	-0.083	0.058	1.52E-01	-0.126	0.054	2.03E-02	-0.294	0.057	2.46E-07
Degree of unsaturation	0.023	0.056	6.79E-01	0.018	0.055	7.44E-01	0.154	0.055	4.94E-03
Glycerides									
Diacylglycerol	0.026	0.074	7.29E-01	-0.013	0.069	8.48E-01	-0.147	0.070	3.49E-02
Triglycerides in HDL	-0.104	0.056	6.42E-02	-0.093	0.053	8.02E-02	-0.211	0.053	7.27E-05
Triglycerides in LDL	-0.060	0.056	2.81E-01	-0.129	0.054	1.75E-02	-0.228	0.055	3.64E-05
Total triglycerides	-0.182	0.057	1.39E-03	-0.131	0.055	1.64E-02	-0.354	0.055	9.77E-11
Total phosphoglycerides	0.014	0.060	8.12E-01	-0.083	0.053	1.19E-01	-0.120	0.058	3.93E-02
Triglycerides in VLDL	-0.192	0.057	7.97E-04	-0.128	0.054	1.89E-02	-0.362	0.055	4.79E-11
Phospholipids									
Phosphatidylcholine	0.033	0.058	5.73E-01	-0.123	0.054	2.32E-02	-0.102	0.058	8.23E-02
Sphingomyelins	0.056	0.055	3.12E-01	-0.060	0.052	2.48E-01	-0.081	0.056	1.47E-01
Total cholines	0.042	0.063	5.05E-01	-0.082	0.058	1.56E-01	-0.134	0.064	3.68E-02
Amino acids									
Alanine	0.027	0.058	6.41E-01	0.083	0.058	1.52E-01	-0.045	0.057	4.25E-01
Glutamine	-0.108	0.057	6.10E-02	-0.047	0.055	3.94E-01	-0.078	0.058	1.81E-01
Histidine	0.048	0.058	4.10E-01	-0.005	0.056	9.25E-01	0.009	0.060	8.74E-01
Isoleucine	-0.112	0.054	3.73E-02	-0.090	0.050	7.23E-02	-0.199	0.051	1.13E-04
Leucine	-0.058	0.050	2.46E-01	-0.088	0.047	6.45E-02	-0.155	0.050	2.13E-03

Phenylalanine	-0.009	0.055	8.67E-01	-0.037	0.053	4.90E-01	-0.018	0.057	7.56E-01
Tyrosine	0.001	0.052	9.85E-01	0.056	0.052	2.80E-01	0.016	0.056	7.76E-01
Valine	0.030	0.050	5.57E-01	-0.007	0.047	8.77E-01	0.001	0.052	9.85E-01
Kidney function									
Albumin	0.099	0.062	1.11E-01	-0.038	0.060	5.32E-01	-0.022	0.058	6.98E-01
Creatinine	-0.006	0.048	9.07E-01	-0.017	0.044	7.00E-01	-0.039	0.048	4.11E-01
Glycolysis									
Citrate	-0.003	0.064	9.65E-01	-0.020	0.061	7.40E-01	-0.020	0.061	7.45E-01
Glucose	0.052	0.053	3.22E-01	-0.008	0.050	8.67E-01	-0.057	0.054	2.99E-01
Lactate	0.048	0.056	3.94E-01	0.084	0.056	1.38E-01	-0.037	0.057	5.25E-01
Inflammation									
Glycoprotein acetyls	-0.154	0.063	1.40E-02	-0.102	0.059	8.43E-02	-0.310	0.059	1.89E-07
Ketone bodies									
Acetate	0.004	0.061	9.42E-01	-0.112	0.057	4.98E-02	-0.120	0.060	4.66E-02
Beta-hydroxybutyrate	-0.009	0.068	8.94E-01	-0.020	0.064	7.57E-01	-0.126	0.066	5.51E-02

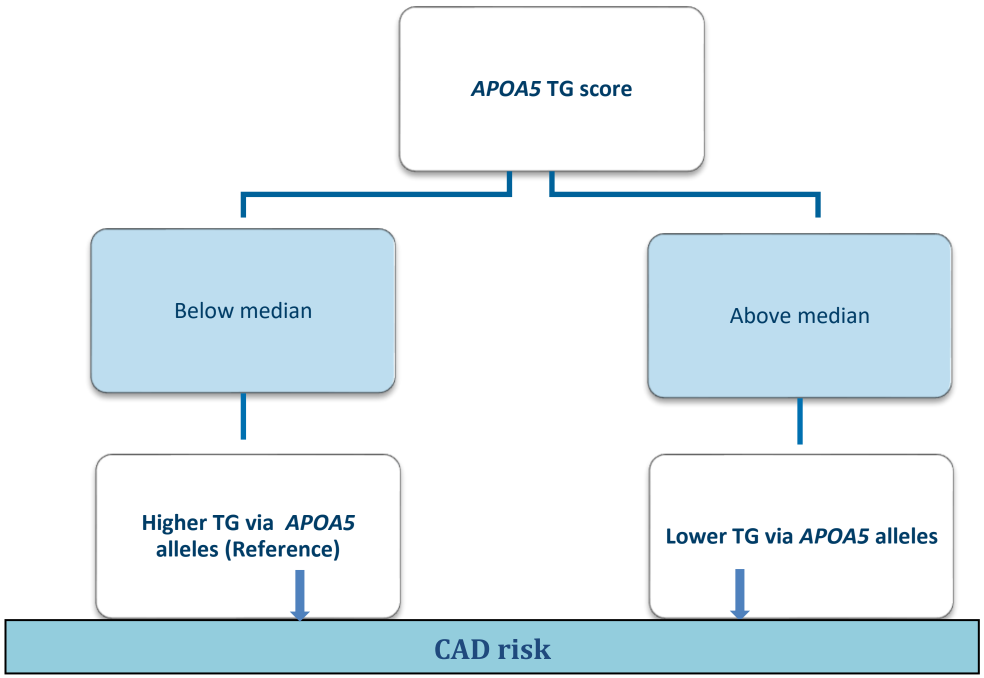

Figure S1 Single instrument genetic analyses of TG-lowering via *APOA5* alleles.
Note. The same design is used for the other two single instrument genetic analyses: TG-lowering via *LPL* alleles; LDL-C-lowering.

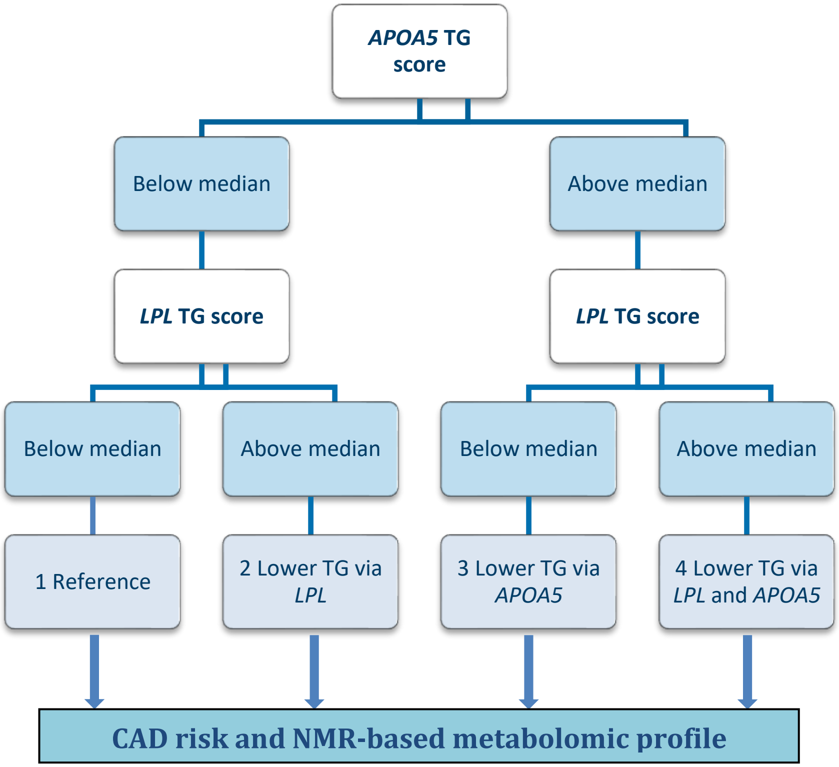

Figure S2 Design of 2 × 2 factorial analyses TG-lowering *APOA5* alleles and TG-lowering *LPL* alleles.

Note. The same design is used for the other 2x2 factorial analyses: TG-lowering *APOA5* alleles and LDL-C-lowering ; TG-lowering LPL alleles and LDL-C-lowering.

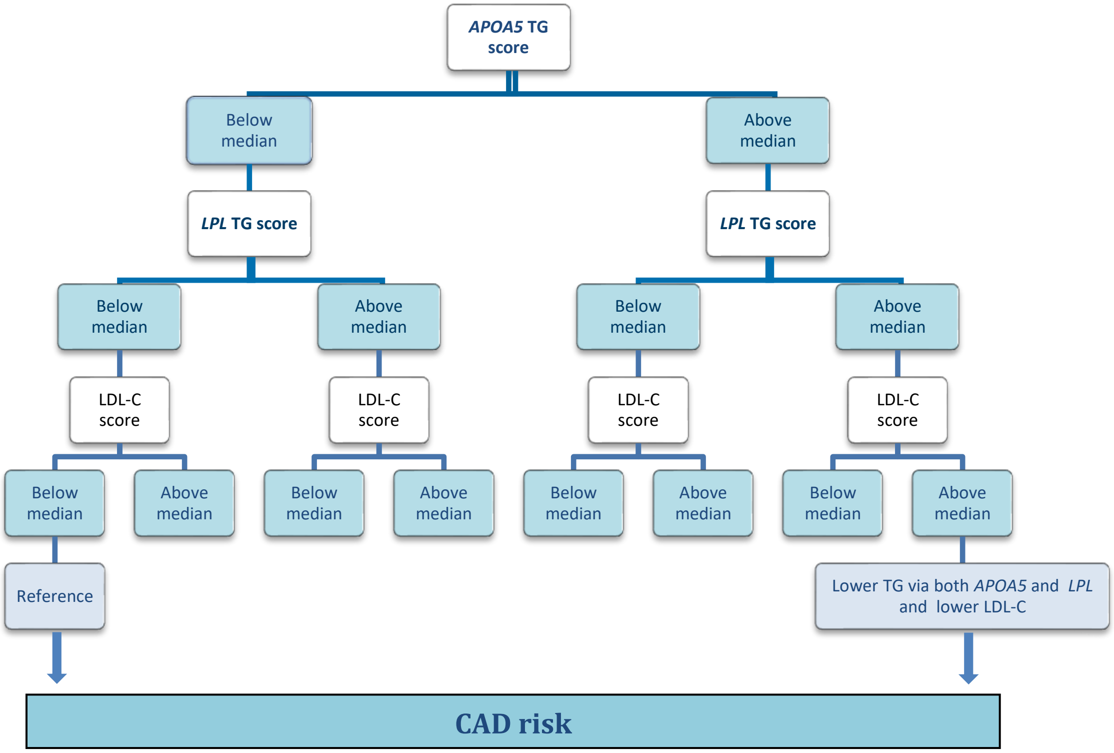

Figure S3 Design of 2 × 2 × 2 factorial analyses of TG-lowering *APOA5* alleles, TG-lowering *LPL* alleles and LDL-C lowering alleles.

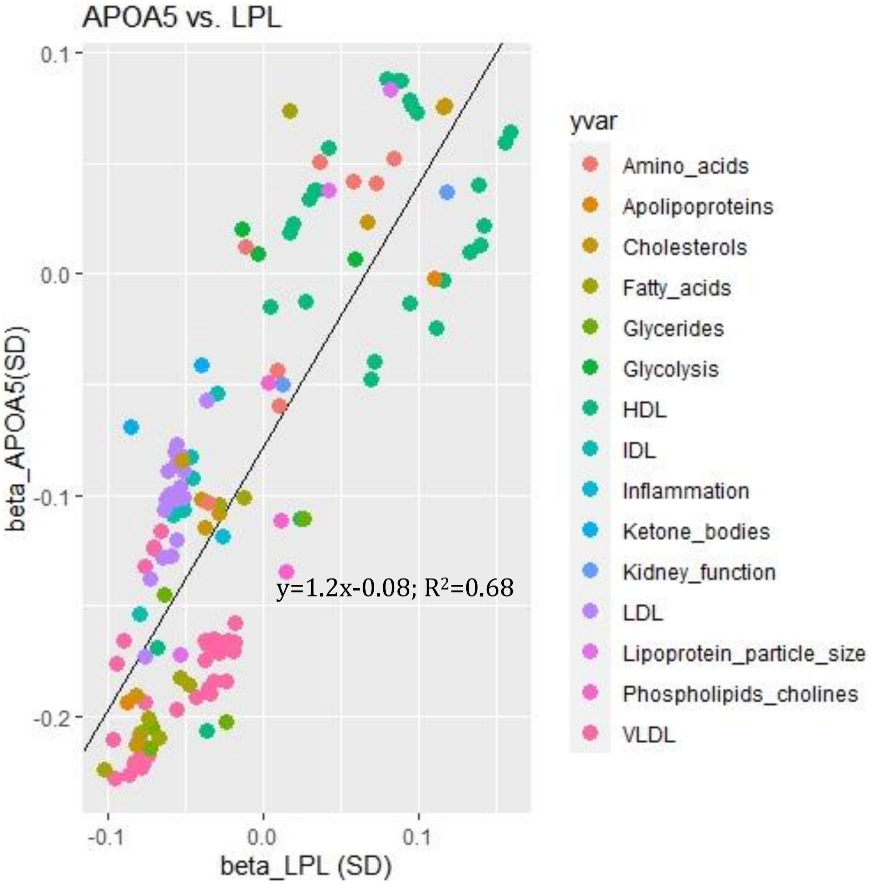

Figure S4 The effect sizes (i.e. beta coefficients) for the associations between genetically-influenced lower TG via *APOA5* and *LPL* and 145 circulating metabolic measures in the Netherlands Epidemiology of Obesity (NEO) and in Oxford Biobank (OBB) cohort.

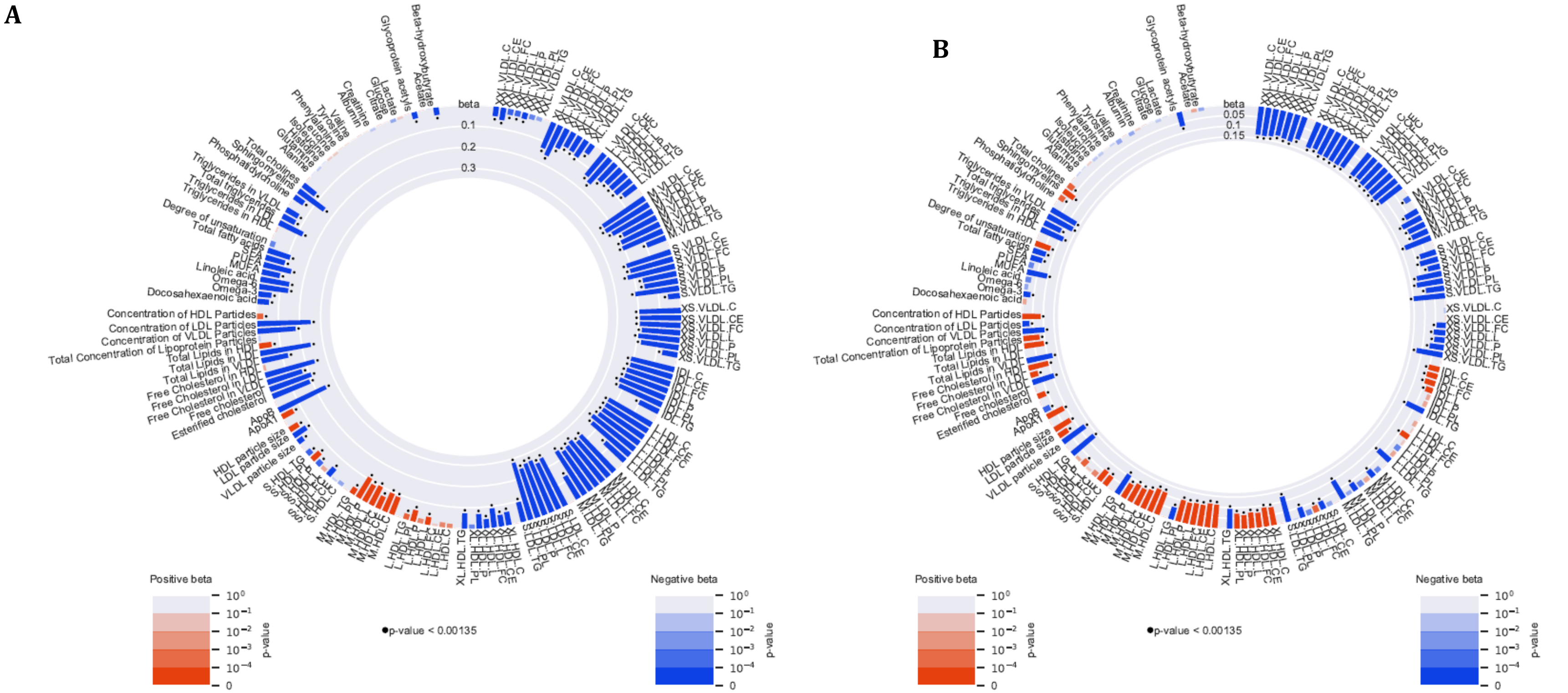

Figure S5. Associations of the genotype group with 145 NMR-based metabolomic measures in factorial analyses in the UK Biobank cohort (UBB) (n = 309,780): **A)** LDL-C-lowering only; **B)** lower TG via *LPL* only; **C)** lower TG via *APOA5* only; **D)** LDL-C-lowering and lower TG via *LPL*; **E)** LDL-C-lowering and lower TG via *APOA5*; **F)** lower TG via both *LPL* and *APOA5*; **G)** all scores combined.

C

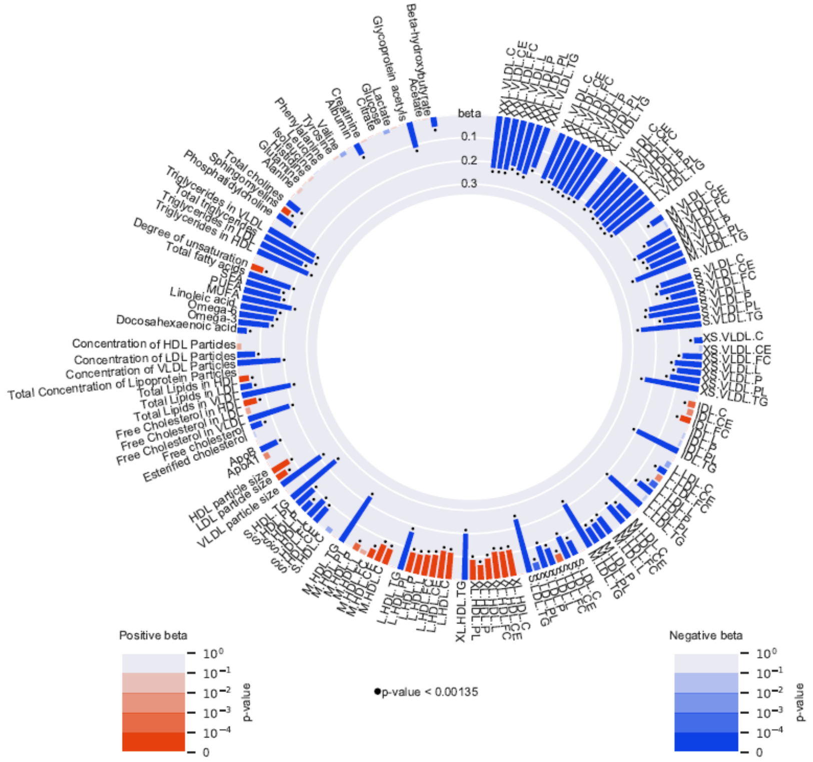

D

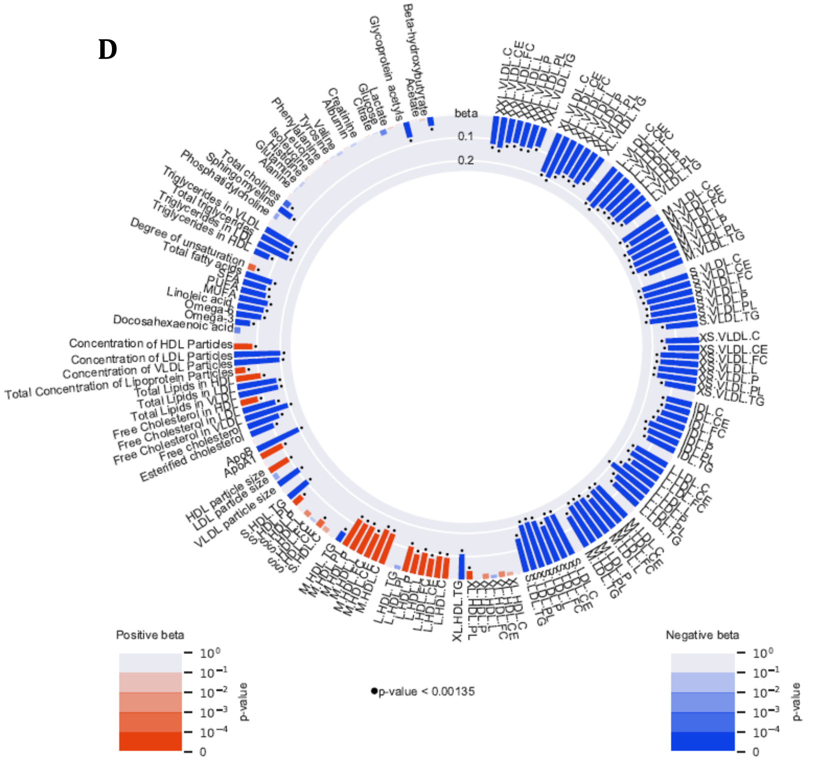

Figure S5 continued.

E

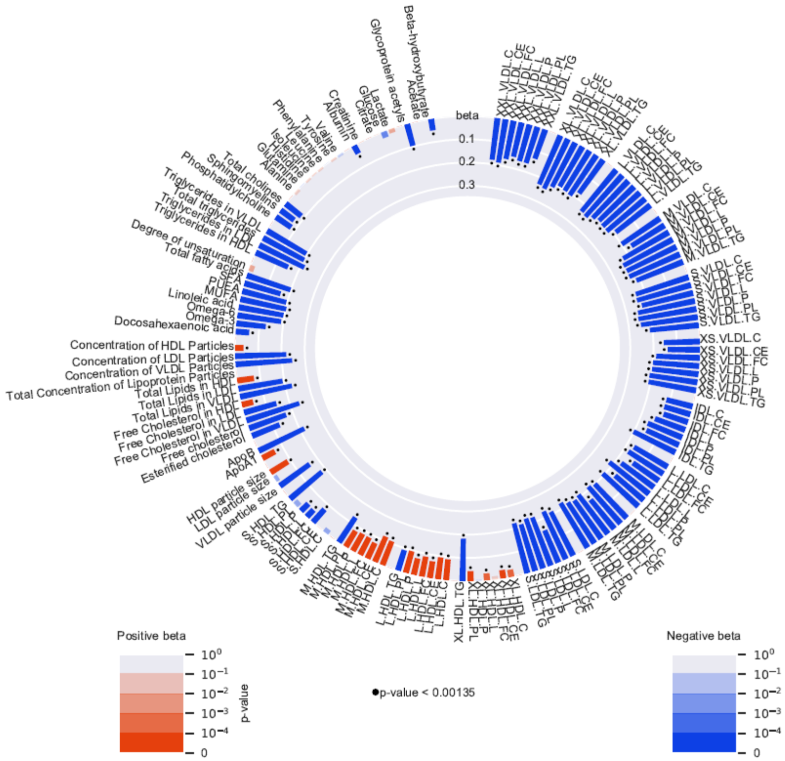
beta
0.1
0.2
0.3
Positive beta
Negative beta
●p-value < 0.00135
p-value

F

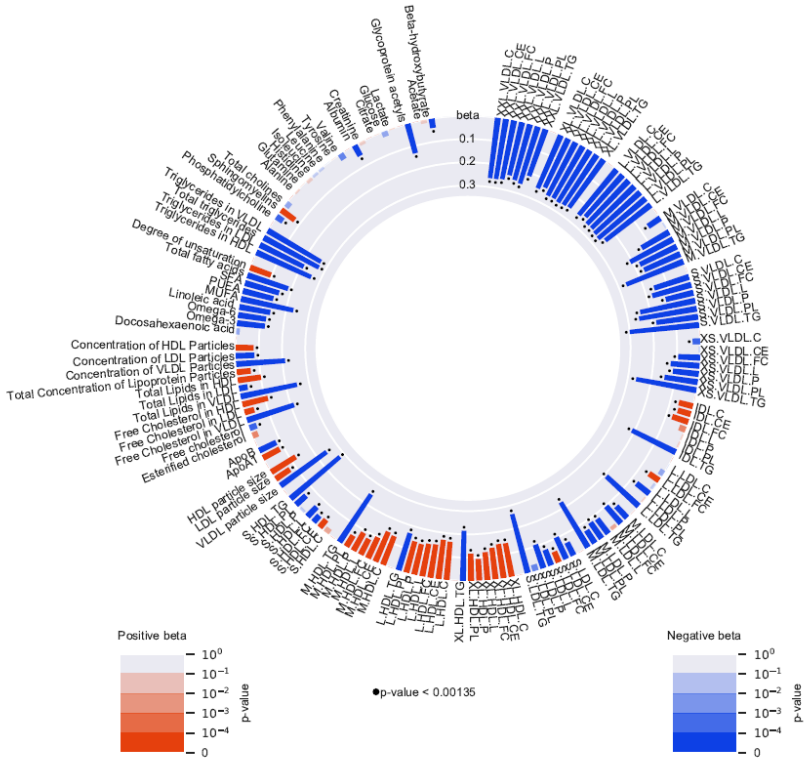
beta
0.1
0.2
0.3
Positive beta
Negative beta
●p-value < 0.00135
p-value

Figure S5 continued.

G

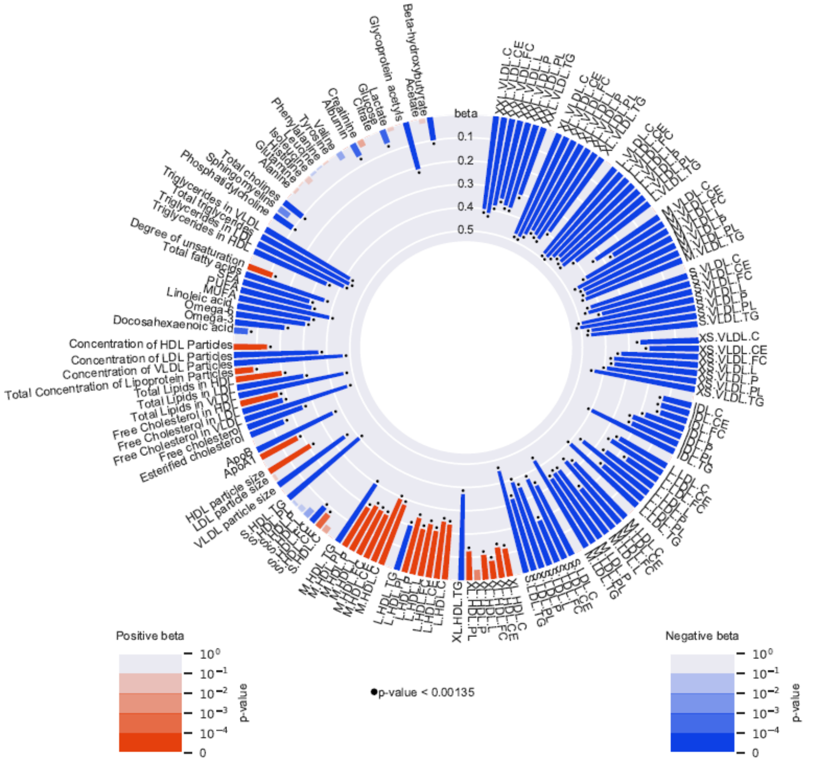

Figure S5 continued.
